# Plasma tissue factor as a promising marker in multiple sclerosis: Evidence from a two-sample Mendelian randomization study

**DOI:** 10.1515/tnsci-2025-0378

**Published:** 2025-08-16

**Authors:** Rui Pan, Aiqi Wang, Yaqi Li, Qizhi Xie, Meihua Lin, Jiayi Li, Xiaolei Shi

**Affiliations:** School of Nursing, Huizhou Health Sciences Polytechnic, Huizhou, Guangdong, P. R. China; School of Pharmacy and Laboratory, Huizhou Health Sciences Polytechnic, Huizhou, Guangdong, P. R. China; The Affiliated Brain Hospital, Guangzhou Medical University, No. 36, Ming Xin Road, Guangzhou, Guangdong, P. R. China; Guangdong Engineering Technology Research Center for Translational Medicine of Mental Disorders, Guangzhou, Guangdong, P. R. China; Key Laboratory of Neurogenetics and Channelopathies of Guangdong Province and the Ministry of Education of China, Guangzhou Medical University, Guangzhou, P. R. China

**Keywords:** coagulation factors, tissue factor, multiple sclerosis, Mendelian randomization

## Abstract

**Background:**

Multiple sclerosis (MS) is a major demyelinating disorder that affects the central nervous system. A growing body of evidence has revealed the involvement of coagulation pathway in the pathogenesis of MS. However, the causal association between coagulation factors and MS is still unclear.

**Method:**

A two-sample Mendelian randomization (MR) analysis was conducted. Genetic variants for plasma coagulation factors were identified as instrumental variables. Summary-level statistics for MS were collected from a large-scale genome-wide association study, including 47,429 cases and 68,374 controls. Primary MR analysis was performed using the inverse-variance weighting (IVW) approach. False discovery rate (FDR)-adjusted method was applied to adjust for multiple testing. MR-Egger, weighted median, simple mode, weighted mode, and MR-pleiotropy residual sum and outlier (MR-PRESSO) methods were used as sensitivity analysis approaches.

**Results:**

A causal effect of higher plasma tissue factor (TF) levels on the risk of MS onset was identified using IVW method (OR: 1.215, 95% CI 1.108–1.333, *P* < 0.001, *P*_FDR_ < 0.001). Complementary analysis using weighted median (OR: 1.262, 95% CI: 1.119–1.423, *P* < 0.001), weighted mode (OR: 1.238, 95% CI: 1.100–1.394, *P* = 0.012), and MR-PRESSO (OR: 1.215, 95% CI: 1.125–1.313, *P* = 0.003) methods yielded consistent results. Null associations were found for other plasma coagulation factors with MS.

**Conclusions:**

The study demonstrates a suggestive association between TF and MS. Increasing plasma TF was associated with an increase in MS risk. TF should be a promising biomarker and new target for MS.

## 1 Introduction

Multiple sclerosis (MS) is a chronic, degenerative disease characterized by multifocal demyelination in the central nervous system (CNS). The pathogenesis of the disorder involves disruption of blood–brain barrier (BBB), extravasation of immunocytes, disseminated neuroinflammation, axonal damage, and demyelination within the brain parenchyma, leading to progressive and irreversible accumulation of physical and cognitive disability [1].

The association between hemostasis, vascular thrombosis, and MS has been taken into consideration since 1882. Ribbert et al. assumed that brain vascular thrombosis caused by hematogenous infection could contribute to MS lesion [2]. Thereafter, Putnam found that venular thrombosis might be the primary cause of MS based on histologic and experimental findings [3]. In addition, most of the MS patients exhibited a peculiar defect in their clotting process, indicating that thrombosis was not a result of vascular injury, but the consequence of hematological changes [4]. Epidemiology studies also observed an increased risk of cardiovascular disease and venous thromboembolism in MS patients [5,6].

As an essential element in the activation of hemostasis, a growing body of evidence has revealed the involvement of the coagulation pathway in the pathogenesis of MS [7,8]. Histopathological analysis demonstrated extensive deposition of coagulation pathway proteins in post-mortem MS plaques [9,10]. Animal models implicate that coagulation factors play a proinflammatory role in experimental allergic encephalomyelitis (EAE), a prototypic model of MS, beyond their hemostatic function [10–12]. Furthermore, clinical studies have shown that higher levels of coagulation factors including factor II (FII), factor X (FX), factor XII (FXII), prothrombin, fibrinogen, and procoagulant microparticles are present in the blood or cerebrospinal fluid (CSF) of MS patients [10,13–15]. Nevertheless, the above findings may be vulnerable to confounding factors, and therefore insufficient to establish reliable causal relationships. It is still necessary to further investigate the causal effects of coagulation components on the risk of MS.

Mendelian randomization (MR) is a genetic epidemiological method used to identify and quantify the causal relationship between clinical traits and disease phenotypes [16,17]. The approach employs genetic instrumental variables (IVs) as substitutes for exposure. By leveraging the random allocation of genetic alleles at conception, MR analysis can effectively avoid the influence of potential confounding, and provide more accurate and credible conclusion [16,17]. Therefore, the present study used a two-sample MR approach to investigate the genetic association between plasma coagulation factors and the risk of MS.

## 2 Materials and methods

### 2.1 Ethical compliance

The study used summary data obtained from publicly available genome-wide association studies (GWASs), which have received approval from the respective institutional review boards.

### 2.2 Study design

**Figure 1:**
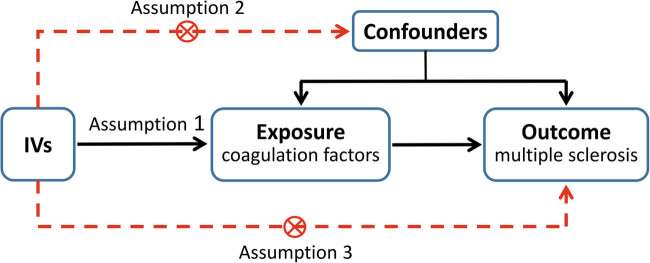
Workflow of MR framework in the study. MR analysis is based on three assumptions. Assumption 1, the chosen genetic variants should demonstrate a strong association with the exposures; Assumption 2, the genetic variants utilized as IVs for the exposures must not be affected by any confounding factors; and Assumption 3, the selected IVs should influence the outcome only via the exposures, rather than through a direct association.

In this work, a univariable two-sample MR analysis was performed to explore the causal relationship between plasma coagulation factors and MS. The study has been structured in accordance with the STROBE-MR guidelines [18]. The successful implementation of our MR analysis is contingent upon three critical assumptions [19]: first, the chosen genetic variants should demonstrate a strong association with the exposures; second, the genetic variants utilized as IVs for the exposures must not be affected by any confounding factors; third, IVs should influence the outcome only via the exposures, rather than through a direct association. The workflow of our MR framework is presented in Figure 1. Moreover, to prevent possible reverse causation, a reverse-MR analysis was conducted.

### 2.3 Data sources

#### 2.3.1 GWAS of plasma coagulation factors

**Figure 2:**
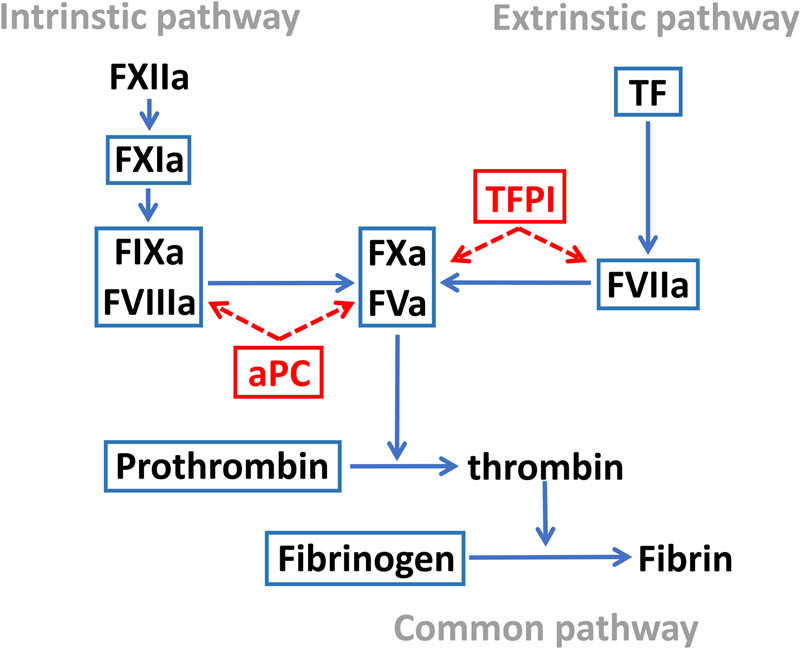
Overview of the coagulation pathways. The hemostatic components investigated in the study are highlighted. TF, tissue factor; FVa, activated factor V; FVIIa, activated factor VII; FVIIIa, activated factor VIII; FIXa, activated factor IX; FXa, activated factor X; FXIa, activated factor XI; FXIIa, activated factor XII; aPC, activated protein C; and TFPI, tissue factor pathway inhibitor.

We selected 11 major coagulation factors which participate in the whole process of intrinsic and/or extrinsic coagulation (Figure 2), including tissue factor (TF), factor V (FV), factor VII (FVII), factor VIII (FVIII), factor IX (FIX), factor X (FX), factor XI (FXI), prothrombin, fibrinogen, protein C (PC), and tissue factor pathway inhibitor (TFPI). Detailed information is summarized in Table 1.

**Table 1:** Detailed information of the studies included in MR analysis

Phenotype	GWAS ID	Sample size (cases/controls)	Number of SNPs	Population	Consortium	Year	Journal	References
MS	ieu-b-18	47,429/68,374	6,304,359	European	IMSGC	2019	Science	Patsopoulos et al. [22]
Plasma TF	ebi-a-GCST90012014	21,758	13,098,661	European	—	2020	Nat Metab	Folkersen et al. [20]
Plasma FV	ebi-a-GCST90019453	10,708	15,567,796	European	—	2020	Nat Commun	Pietzner et al. [23]
Plasma FVII	ebi-a-GCST90019432	10,708	15,567,356	European	—	2020	Nat Commun	Pietzner et al. [23]
Plasma FVIII	ebi-a-GCST90019386	10,708	15,568,124	European	—	2020	Nat Commun	Pietzner et al. [23]
Plasma FIX	ebi-a-GCST90019451	10,708	15,567,881	European	—	2020	Nat Commun	Pietzner et al. [23]
Plasma FX	ebi-a-GCST90019452	10,708	15,568,033	European	—	2020	Nat Commun	Pietzner et al. [23]
Plasma FXI	ebi-a-GCST90019413	10,708	15,568,193	European	—	2020	Nat Commun	Pietzner et al. [23]
Plasma prothrombin	ebi-a-GCST90019459	10,708	15,567,507	European	—	2020	Nat Commun	Pietzner et al. [23]
Plasma fibrinogen	ebi-a-GCST90019421	10,708	15,567,759	European	—	2020	Nat Commun	Pietzner et al. [23]
Plasma PC	ebi-a-GCST90019423	10,708	15,567,760	European	—	2020	Nat Commun	Pietzner et al. [23]
Plasma TFPI	prot-a-2957	3,301	10,534,735	European	—	2018	Nature	Sun et al. [21]

Abbreviations: TF, tissue factor; F(V, VII, VIII, IX, X, XI), factor (V, VII, VIII, IX, X, XI); PC, protein C; TFPI, tissue factor pathway inhibitor; IMSGC, International Multiple Sclerosis Genetics Consortium.

Genetic instruments for plasma TF levels were derived from a large-scale genome-wide meta-analysis involving 13 European-ancestry cohorts [20]. The study included 21,758 individuals, with protein levels measured using the Olink Proximity Extension Assay CVD-I panel. Summary statistics were generated through standardized imputation (1000 Genomes Project phase 3 or later, or Haplotype Reference Consortium) and quality control (call rate filters, sex mismatches, population outliers, heterozygosity deviations, and cryptic relatedness). To avoid potential batch-differences between cohorts, protein measurements (NPX values) were rank-based inverse normal transformed and standardized to unit variance. Genetic analyses employed additive linear regression models adjusted for population structure and study-specific parameters. To ensure robustness, meta-analysis was performed in duplicate at two independent research centers using separate bioinformatic.

Genetic variants for plasma TFPI were extracted from a GWAS dataset, including 3,301 European individuals from the INTERVAL study [21]. Participants were recruited between 2012 and 2014 at 25 National Health Service Blood and Transplant centers across England. Standardized online questionnaires were used to capture demographic characteristics (age, sex, ethnicity), anthropometric measurements (height, weight), and lifestyle factors (alcohol consumption, smoking status, diet). Inclusion criteria required participants to be aged ≥18 years without a history of major comorbidities (e.g., cardiovascular disease, cancer, chronic infections) or recent acute illness. From the original INTERVAL cohort of about 50,000 individuals, two non-overlapping subcohorts (*n* = 2,731 and *n* = 831) were randomly selected. After genetic quality control, 3,301 participants (2,481 and 820 from each subcohort) were retained for analysis. Plasma TFPI levels were measured using a multiplexed, aptamer-based approach (SOMAscan assay). Genetic association analyses were performed in SNPTEST v2.5.2 by applying an additive genetic model through simple linear regression.

For the rest hemostatic components (FV, FVII, FVIII, FIX, FX, FXI, prothrombin, fibrinogen, and PC), summary-level data were obtained from a Fenland study [23], which is a population-based cohort conducted in the Cambridgeshire region of the United Kingdom. The recruitment occurred between 2005 and 2015. Exclusion criteria were diabetes mellitus, mobility impairment, terminal illness, psychiatric disorders, or current pregnancy/lactation. After excluding ancestry outliers and related individuals, the final GWAS analysis included 10,708 participants (mean age 48.6 ± 7.5 years; 53.4% female). Proteomic measurements underwent rank-based inverse normal transformation, followed by adjustment for age, sex, sample collection site, and ten genetic principal components. Genome-wide analyses were performed using BGENIE v1.3.

#### 2.3.2 GWAS of MS

Summary-level data for MS were extracted from the International Multiple Sclerosis Genetics Consortium (IMSGC), which compared 47,429 MS patients (including the relapsing-remitting and the progressive form) and 68,374 non-MS controls of European ancestry [22]. As of the end of 2011, the IMSGC analyzed all available to the IMSGC GWAS data and two other datasets, one from Rotterdam, Netherlands (Rotterdam) and another one from US (Berkeley; Kaiser Permanente). The IMSGC GWAS data included six datasets from Patsopoulos et al. [24] and data from the WTCCC2 and IMSGC [25] studies. MS cases of the Rotterdam study were recruited through a nationwide study in the Netherlands. Diagnoses were confirmed at the Rotterdam MS Centre ErasMS outpatient clinic. In the Berkeley study, cases and controls were recruited from the Kaiser Permanente Medical Care Plan (Northern California Region). Eligible MS patients were aged 18–69 years. Diagnoses were validated via electronic health record review and structured interviews. The majority of the participants included in these datasets were of European descent. All the MS patients were diagnosed based on the standard diagnostic criteria [26,27].

As described, the GWAS data for both exposures and outcomes were obtained from independent cohorts, thereby minimizing the potential impact of sample overlap on the results. Furthermore, as all samples comprised individuals of European ancestry, it is reasonable to assume that the genetic variant-exposure associations were similar between the exposure and outcome cohorts.

### 2.4 Selection of instruments

A series of processes were performed to extract eligible IVs. First, single-nucleotide polymorphisms (SNPs) that exhibited a *P*-value of genome-wide significance were chosen as the potential IVs for exposures. When the threshold for genome-wide significance was set at 5 × 10^−8^, only two SNPs for FVII, prothrombin, fibrinogen, and PC and one SNPs for FIX and TFPI were extracted. Then the criteria were relaxed to 1 × 10^−5^ to include more SNPs for analysis. For the rest coagulation factors (including TF, FV, FVIII, FX, and FXI), the threshold was set as 5 × 10^−8^ (Table S2). Second, SNPs that were in linkage disequilibrium (LD; *r* ^2^ threshold <0.001 within a 10 Mb window) were excluded to avoid bias. The retained SNPs were extracted from the outcome GWAS dataset (IMSGC). The associations between SNPs and outcomes (e.g., *β* and *P*-values) utilized in our analysis were directly extracted from the original GWAS summary statistics. These associations had been previously computed by comparing allele frequencies between MS cases and control groups. In addition, proxy SNPs in LD (*r* ^2^ > 0.8) were used to replace the instrumental SNPs that were absent from the outcome dataset. Third, in order to avoid weak instrumental bias, the strength of each SNPs was assessed by calculating *F*-statistics. An *F*-statistic >10 indicated that the SNP was robust enough for MR analysis [28]. The *F*-statistic was calculated for each SNP using the formula: *F*-statistic = *R* ^2^ × (*N* − 2)/(1 − *R* ^2^), where *R* ^2^ was calculated according to the formula: *R* ^2^ = 2 × EAF × (1 − EAF) × *β* ^2^, where *N* is the sample size of the exposure GWAS, EAF is the effect allele frequency, and *β* is the estimated effect on the exposure, representing the effect of each allele on standardized plasma protein levels (units: standard deviation, SD). The effect size (*β*) of each SNP on the exposure was obtained directly from the summary statistics of the respective GWAS datasets. Finally, we manually screened the chosen SNPs using the PhenoScanner database (Version 2, http://www.phenoscanner.medschl.cam.ac.uk/) [29] and the LDlink web tool (https://ldlink.nih.gov/) to identify other traits that may affect the outcome, with a genome-wide significant level of *P* < 5 × 10^−8^. A related SNP that was directly associated with MS has been removed from the MR analysis (Table S1).

### 2.5 Statistical analysis

The primary MR analysis was performed using inverse-variance weighting (IVW) approach [30]. Several alternative methods were conducted as sensitivity analysis, including weighted median, weighted mode, simple mode, MR-Egger, and MR-pleiotropy residual sum and outlier (MR-PRESSO) [31,32]. A *P*-value less than 0.05 was considered to be statistically significant. To adjust for multiple testing, Benjamini-Hochberg method was used to calculate false discovery rate (FDR)-corrected *P*-values. Furthermore, Cochran’s *Q*-test and leave-one-out analysis were conducted to assess the heterogeneity across the SNPs [33]. The MR-PRESSO global test, MR-Egger intercept test, and visual inspection of the funnel plot were performed to identify potential horizontal pleiotropy [34]. A *P*-value of <0.05 suggested that the IVW results might not be valid due to the existence of heterogeneity or pleiotropy.

All the analyses were conducted using the TwoSampleMR [35] and MR-PRESSO [34] packages in R software (version 4.1.2).

## 3 Results

### 3.1 Associations of plasma TF levels with MS

Seven SNPs significantly associated with plasma TF level were extracted. The *F*-statistics for all SNPs ranged from 41 to 455, suggesting a low probability for weak instrumental bias. Detailed information of the included SNPs is available in Table S2.

**Figure 3:**
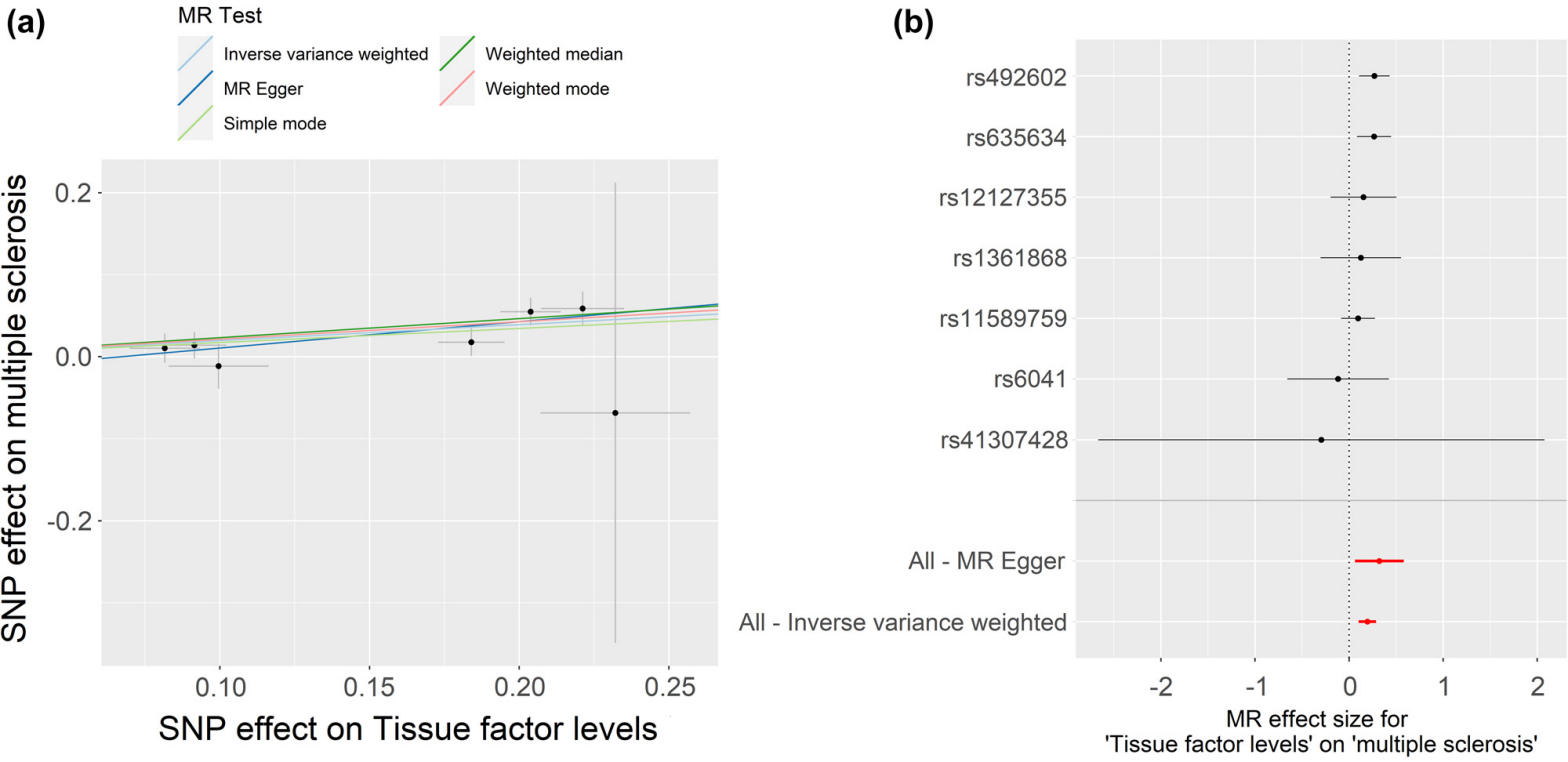
Scatter plot (a) and forest plot (b) of the causal effect of plasma TF on MS risk. TF, tissue factor; MS, multiple sclerosis.

**Figure 4:**
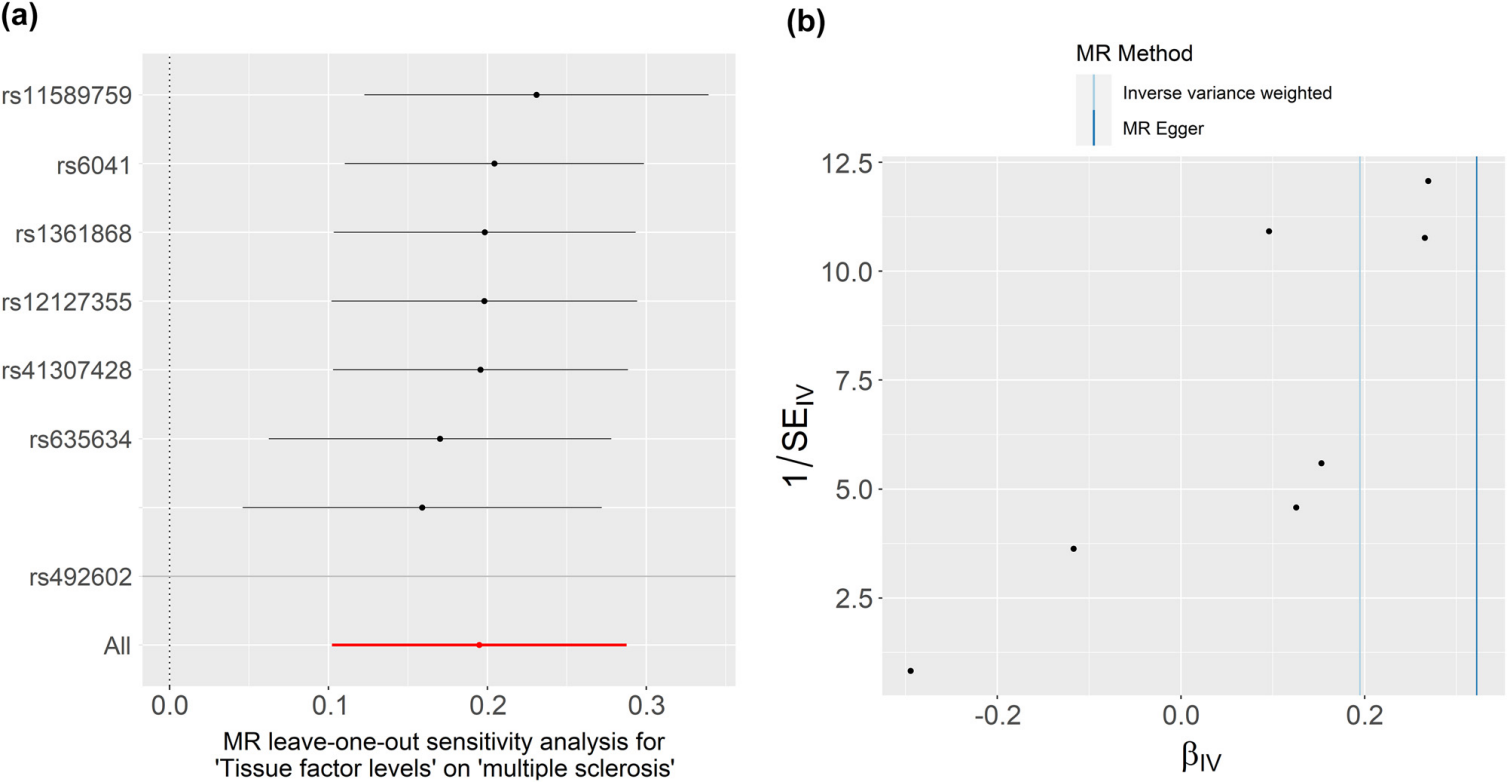
Leave-one-SNP-out sensitivity analysis (a) and funnel plot (b) for plasma TF on MS risk. TF, tissue factor; MS, multiple sclerosis.

The results of IVW model suggested that each SD increase in plasma TF was associated with an elevated risk of MS (OR: 1.215, 95% CI: 1.108–1.333, *P* < 0.001, *P*_FDR_ < 0.001) (Table 2). The scatter plot and forest plot are shown in Figure 3. Sensitivity analysis using weighted median (OR: 1.262, 95% CI: 1.124–1.416, *P* < 0.001), weighted mode (OR: 1.238, 95% CI: 1.093–1.402, *P* = 0.015), and MR-PRESSO (OR: 1.215, 95% CI: 1.125–1.313, *P* = 0.003) yielded consistent results (Table 3). Cochran’s *Q*-test demonstrated no significant heterogeneity (*Q*-value = 4.149, *P*_*Q*_ = 0.657). The MR-Egger intercept test indicated no horizontal pleiotropy (intercept = −0.022, *P*_intercept_ = 0.351). The MR-PRESSO global test also failed to reveal any potential pleiotropy and outliers (*P* = 0.646) (Table 4). In addition, the leave-one-out analysis was conducted for SNP conformity. Our IVW results were not affected by any single SNP (Figure 4a). The funnel plot is presented in Figure 4b.

**Table 2:** Main results of MR analysis

Outcome	Exposure	nSNPs	Method	OR (95% CI)	*P-*value	*P*_FDR_
MS	Plasma TF	7	IVW	1.215 (1.108–1.333)	**<0.001**	**<0.001**
	Plasma FV	4	IVW	1.093 (0.593–2.014)	0.777	0.844
	Plasma FVII	18	IVW	1.018 (0.945–1.096)	0.642	0.844
	Plasma FVIII	7	IVW	0.958 (0.890–1.031)	0.249	0.548
	Plasma FIX	15	IVW	0.978 (0.860–1.112)	0.730	0.844
	Plasma FX	6	IVW	1.119 (0.962–1.302)	0.144	0.548
	Plasma FXI	4	IVW	0.993 (0.947–1.041)	0.773	0.844
	Plasma prothrombin	11	IVW	1.147 (0.931–1.413)	0.197	0.548
	Plasma fibrinogen	17	IVW	1.013 (0.893–1.148)	0.844	0.844
	Plasma PC	21	IVW	1.078 (0.958–1.213)	0.213	0.548
	Plasma TFPI	17	IVW	1.032 (0.940–1.133)	0.511	0.844

Bold symbol indicated statistically significances (*P* < 0.05). Abbreviations: TF, tissue factor; F(V, VII, VIII, IX, X, XI), factor (V, VII, VIII, IX, X, XI); PC, protein C; TFPI, tissue factor pathway inhibitor; IVW, inverse-variance weighted; nSNPs, number of single-nucleotide polymorphisms; OR, odds ratio; CI, confidence interval; FDR, false discovery rate.

**Table 3:** Sensitivity analysis of the associations between circulating hemostasis components with MS

Outcome	Exposure	MR-Egger	Weighted median	Simple mode	Weighted mode	MR-PRESSO
		OR (95% CI)	OR (95% CI)	OR (95% CI)	OR (95% CI)	OR (95% CI)
		*P*-value	*P*-value	*P*-value	*P*-value	*P*-value
MS	Plasma TF	1.380 (1.065–1.788)	1.262 (1.124–1.416)	1.188 (1.015–1.390)	1.238 (1.093–1.402)	1.215 (1.125–1.313)
		0.059	**<0.001**	0.075	**0.015**	**0.003**
	Plasma FV	0.249 (0.000–3570.452)	1.046 (0.896–1.221)	1.057 (0.908–1.231)	1.046 (0.898–1.218)	1.108 (0.667–1.839)
		0.803	0.567	0.528	0.606	0.712
	Plasma FVII	1.057 (0.964–1.158)	1.042 (0.966–1.124)	1.051 (0.807–1.369)	1.043 (0.966–1.125)	1.016 (0.973–1.060)
		0.256	0.285	0.717	0.297	0.478
	Plasma FVIII	0.921 (0.831–1.020)	0.958 (0.890–1.002)	0.982 (0.805–1.199)	0.941 (0.888–0.997)	0.958 (0.890–1.031)
		0.173	0.058	0.867	0.087	0.293
	Plasma FIX	1.067 (0.733–1.552)	0.909 (0.764–1.083)	0.858 (0.656–1.122)	0.855 (0.703–1.039)	0.989 (0.903–1.082)
		0.740	0.285	0.281	0.138	0.806
	Plasma FX	1.057 (0.802–1.394)	1.105 (0.965–1.266)	1.089 (0.899–1.319)	1.108 (0.960–1.279)	1.098 (0.964–1.250)
		0.714	0.150	0.423	0.219	0.201
	Plasma FXI	1.015 (0.870–1.184)	0.999 (0.950–1.051)	0.996 (0.925–1.073)	1.002 (0.953–1.054)	0.993 (0.971–1.015)
		0.870	0.977	0.926	0.935	0.578
	Plasma prothrombin	1.788 (0.910–3.514)	1.284 (1.019–1.617)	1.333 (0.944–1.882)	1.339 (1.018–1.761)	1.172 (0.965–1.424)
		0.126	**0.034**	0.133	0.063	0.138
	Plasma fibrinogen	0.924 (0.665–1.283)	1.010 (0.847–1.205)	1.137 (0.816–1.584)	0.814 (0.642–1.033)	1.013 (0.893–1.148)
		0.642	0.910	0.460	0.109	0.846
	Plasma PC	0.981 (0.702–1.372)	1.069 (0.913–1.251)	1.089 (0.816–1.453)	1.086 (0.837–1.408)	1.031 (0.980–1.084)
		0.913	0.408	0.571	0.544	0.247
	Plasma TFPI	1.072 (0.751–1.532)	1.036 (0.920–1.166)	1.037 (0.864–1.244)	1.037 (0.885–1.215)	1.027 (0.952–1.108)
		0.707	0.558	0.705	0.663	0.505

Bold symbol indicated statistically significances (*P* < 0.05). Abbreviations: TF, tissue factor; F(V, VII, VIII, IX, X, XI), factor (V, VII, VIII, IX, X, XI); PC, protein C; TFPI, tissue factor pathway inhibitor; MR-PRESSO, pleiotropy residual sum and outlier; OR, odds ratio; CI, confidence interval.

**Table 4:** Heterogeneity and pleiotropy tests for the associations of circulating hemostasis components with MS

Outcome	Exposure	Cochrane’s *Q*-test		MR-Egger intercept test		MRPRESSOglobal test
		*Q*-value	*P*_*Q*_	Intercept	*P*_intercept_	*P*-value
MS	Plasma TF	4.149	0.657	−0.022	0.351	0.646
	Plasma FV	76.443	**<0.001**	0.264	0.790	**<0.001**
	Plasma FVII	20.693	0.240	−0.010	0.207	0.584
	Plasma FVIII	11.727	0.068	0.014	0.331	0.364
	Plasma FIX	14.578	0.408	−0.011	0.633	0.579
	Plasma FX	8.857	0.115	0.010	0.641	0.195
	Plasma FXI	0.645	0.886	−0.010	0.800	0.808
	Plasma prothrombin	17.645	0.061	−0.037	0.210	0.069
	Plasma fibrinogen	18.479	0.297	0.009	0.560	0.264
	Plasma PC	9.522	0.976	0.008	0.564	0.992
	Plasma TFPI	22.717	0.121	−0.006	0.829	0.170

Bold symbol indicated statistically significances (*P* < 0.05). Abbreviations: TF, tissue factor; F(V, VII, VIII, IX, X, XI), factor (V, VII, VIII, IX, X, XI); PC, protein C; TFPI, tissue factor pathway inhibitor; MR-PRESSO, pleiotropy residual sum and outlier.

### 3.2 Associations of other plasma hemostasis components levels with MS

No significant results were found for the causal association of other plasma hemostasis components with MS (Table 2). The scatter plot, forest plot, leave-one-out plot, and funnel plot are shown in Figures S1–S10.

### 3.3 Reverse-MR analysis

To investigate potential reverse causality, a reverse-MR analysis was carried out. In the initial screening using a genome-wide significance threshold of 5 × 10^−8^, no SNPs associated with MS met this criterion. Therefore, we relaxed the significance threshold to 1 × 10^−7^, which enabled the identification of candidate SNPs. To exclude the influence of confounding factors on causal effects, three SNPs that demonstrated direct associations with coagulation factors or venous thromboembolic disease were excluded (Table S1). Then, 64 SNPs were chosen as IVs for the reverse-MR analysis (Table S3). The results revealed no evidence of causal effects of MS on coagulation factor levels across all methodological approaches (Table S4).

## 4 Discussion

Coagulation factors are well known for their role in the coagulation cascade and hemostasis. In recent years, increasing evidence suggests that the coagulation pathway may also be involved in the pathogenesis of MS [7,8]. The study evaluated the associations between a range of genetically predicted circulating hemostasis components and MS, using a two-sample MR method. Our results show that an increase of one SD in genetically determined plasma TF was associated with a 21.5% increase of the risk of MS (IVW, OR: 1.215, 95% CI: 1.108–1.333). The robustness of this association is further substantiated by the consistency of causal estimates obtained through multiple MR methods, including the weighted median, weighted mode, and MR-PRESSO approaches. Furthermore, the reverse MR analyses show no causal effect of MS on TF levels (OR: 0.995, 95% CI: 0.978–1.013, *P* = 0.592). These findings collectively suggest a causal relationship between elevated plasma TF and the risk of MS onset.

As the key trigger of the extrinsic coagulation cascade for arresting bleeding, TF is highly expressed in the subendothelial tissue [36]. Traditionally, vascular injury induces the release of subendothelial TF. This exposed TF binds to circulating FVII to form the activated TF:FVIIa complex. Then, the TF:FVIIa complex activates FX and FV, and ultimately leads to thrombin generation, fibrin deposition, and physiological haemostasis. Clinical investigations have demonstrated increased concentrations of various coagulation factors (e.g., FII, FX, FXII, prothrombin, and fibrinogen) in the blood or CSF of individuals diagnosed with MS [10,13–15]. In particular, a recent clinical study compared serum/plasma levels of complement/coagulation/vascular factors, viral/microbiological assays, fat-soluble vitamins, and lymphocyte count among individuals with MS during periods of clinical remission or relapse and age/sex-matched controls [37]. The researchers (Tatiana et al.) built two predictive models to determine the most specific pro-coagulative/vascular factor for MS. In both models, the systemic level of TF emerged as a crucial variable in effectively discriminating either MS from controls or MS relapse from remission. The observational finding seemed to be consistent with our MR results suggesting the involvement of TF in MS pathogenesis. However, it should be noted that Tatiana et al. paradoxically observed a significant reduction of circulating TF levels in MS patients compared to healthy controls. Notably, this reduction was even more pronounced during relapse phases compared to clinical remission. Tatiana et al. speculated that the detected TF was free, unbound in complex form. In the pathological state of MS, most of the circulating TF combined with FVII to form a TF:FVIIa complex, resulting in the reduced levels of free TF observed in plasma. This phenomenon mirrors the “consumption” of clotting factors during thrombotic events, where active complexes deplete free circulating components. Notably, our MR analysis demonstrates that elevated TF (as proxied by genetic variants) leads to an increased risk of MS development. The MR approach, which is based on genetic variants, could effectively circumvent potential confounding factors arising from disease-induced alterations TF molecular forms (such as free TF versus TF:FVIIa complexes). Consequently, it enables an assessment of the etiological relationship between genetically determined baseline TF levels and the pathogenesis of MS, independent of secondary changes during disease activity. The genetic predisposition to higher TF levels may contribute to TF:FVIIa complex formation and disease development, while subsequent disease activity alters the circulating form of TF.

The role of TF in MS pathogenesis may be explained by the “infection – immunothrombosis – neuroinflammation” framework [38,39]. Studies have shown that low levels of TF can also be detected in a cryptic state on blood cells including monocytes, neutrophils, and platelets [40,41]. Additionally, TF can also be found in a circulating form of TF-bearing microparticles [42]. Chronic/recurrent infections (a risk factors for MS) may activate the coagulation pathway via circulating TF, leading to the formation of immunothrombosis [43–45]. While this process serves as an evolutionary defense mechanism to restrict the spread of infections and facilitate the elimination of pathogens [46], the hypercoagulable state could result in neuropathological consequences. In addition to vascular obstruction, immunothrombosis could also activate various inflammatory mediators, including cytokines and chemokines. These signaling molecules recruit inflammatory cells, which then migrate toward the CNS. The cellular infiltration further disrupts the BBB [39,47], which is a critical pathological feature in MS development [48,49]. The compromised BBB allows coagulation factors to enter the CNS environment, and initiate a cascade of pathological processes that exacerbate disease progression.

In the CNS, TF has also been reported to be expressed by astrocytes [50]. Disruption of the BBB allows TF to interact with circulating coagulation factors, activating the extrinsic coagulation pathway and leading to sustained thrombin generation [51]. Thrombin is a pivotal serine protease that has been reported to exacerbate neuroinflammation through protease-activated receptors (PARs). Specifically, thrombin-induced activation of PAR initiates a pro-inflammatory cascade in endothelial cells and glial cells [14,15]. Activated microglia release pro-inflammatory cytokines (e.g., IL-1β, TNF-α) that amplify immune cell recruitment. Simultaneously, PAR signaling in endothelial cells compromises the integrity of the BBB through cytoskeletal rearrangement and increased vascular permeability, which in turn facilitates the infiltration of leukocytes. Furthermore, as the terminal product of coagulation cascade, fibrin could also promote the activation and proliferation of resident innate immune cells in CNS [52]. Through binding to the CD11b/CD18 integrin receptor, fibrin is able to prompt microglia and astrocytes to release pro-inflammatory cytokines and chemokines [53,54]. These processes ultimately result in axonal damage, demyelination, and neurodegeneration [55]. Indeed, a series of coagulation-related proteins (e.g., TF, fibrin, and PC inhibitor), have been observed in chronic active MS plaques [52,56]. It has also been reported that fibrin deposition can be observed even before the onset of clinical symptoms [57]. The expression of these coagulation proteins likely indicates the activation of the TF-associated coagulation cascade within MS lesions, thereby contributing to inflammation and tissue damage.

In animal model of EAE, researchers have demonstrated that the administration of thrombin inhibitors (hirudin) and fibrin(ogen)-targeted therapies (Batroxobin) can suppress the production of pro-inflammatory cytokines and mitigate the severity of the disease [10,58,59]. Nonetheless, our MR analysis did not identify any causal association between prothrombin/thrombin and fibrinogen/fibrin, as well as other components (FVII, FX, and FV) of the extrinsic coagulation pathway, and the risk of MS. Our genetic evidence suggests that the alteration of these coagulation components is a secondary consequence of TF-driven coagulation activation, rather than primary causal contributors. These findings provide additional evidence that TF, as the initiator of the extrinsic coagulation cascade, constitutes a critical genetic risk factor in the pathogenesis of MS. In contrast, downstream factors may primarily represent the biochemical outcomes of this activation. Future research should prioritize the development of therapeutic strategies that specifically target TF, rather than broadly suppressing downstream components of the coagulation cascade. This approach may offer a safer and more effective therapeutic strategy for the treatment of MS.

Our MR analysis did not find any causal relationship between intrinsic coagulation components (FXI, FIX, FVIII, and aPC) and the risk of MS. We speculated that the effects of these intrinsic coagulation factors might rely on the intrinsic pathway promoter FXII. Similar to TF, FXII may be another decisive genetic risk factor for MS pathogenesis. Unfortunately, we could not clarify this relationship due to the lack of available GWAS data for FXII. Additional research is required to validate our findings.

A notable advantage of the study is that relatively large GWAS data sources were used to examine the effects of coagulation factors on MS. Another strength of the study is that we identified almost all extrinsic and intrinsic pathway factors to ensure the understanding of the role of coagulation system in MS under MR frameworks. Inevitably, there were also some limitations in this work. First, the utilized GWAS datasets were not stratified by MS subtypes, which precluded us from investigation of whether similar findings could be found in different MS populations. Second, due to the limited understanding of individual data and adjusting factors, the study failed to carry out a comprehensive assessment of the associations between individual genetic variants and confounding factors. Third, the majority of the GWASs data used in the study were derived from European population, which could hinder the generalization of the findings to other racial/ethnic populations.

## 5 Conclusions

In conclusion, the present study demonstrates that genetic predisposition to plasma TF is associated with the risk of MS onset. TF appears to be a promising biomarker for MS diagnosis and prognosis, and a possible new target for future therapeutic strategies of MS.

## Supplementary Material

Supplementary material
