# Supplementary material for: Plasma tissue factor as a promising marker in multiple sclerosis: Evidence from a two-sample Mendelian randomization study
Source: Transl Neurosci. 2025 Aug 16;16(1):20250378. doi: 10.1515/tnsci-2025-0378 (PMC12413626; doi:10.1515/tnsci-2025-0378)
Supplement: Supplementary material [file tnsci-2025-0378-sm.pdf]

Supplementary material

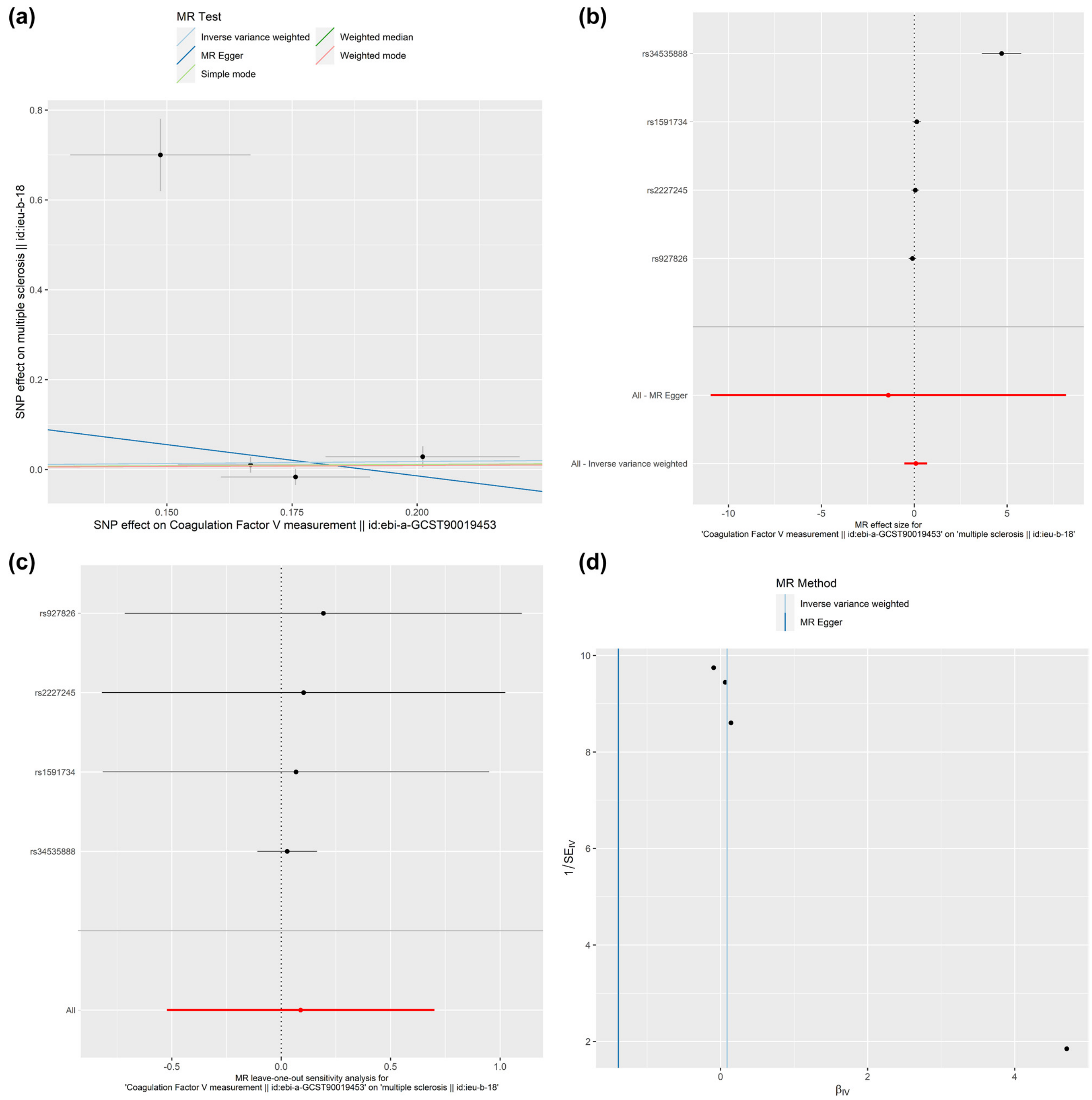

Figure S1: The causal effect of plasma FV on MS risk. (a) Scatter plot, (b) Forest plot, (c) Leave one out plot and (d) Funnel plot. FV, factor V; MS, multiple sclerosis.

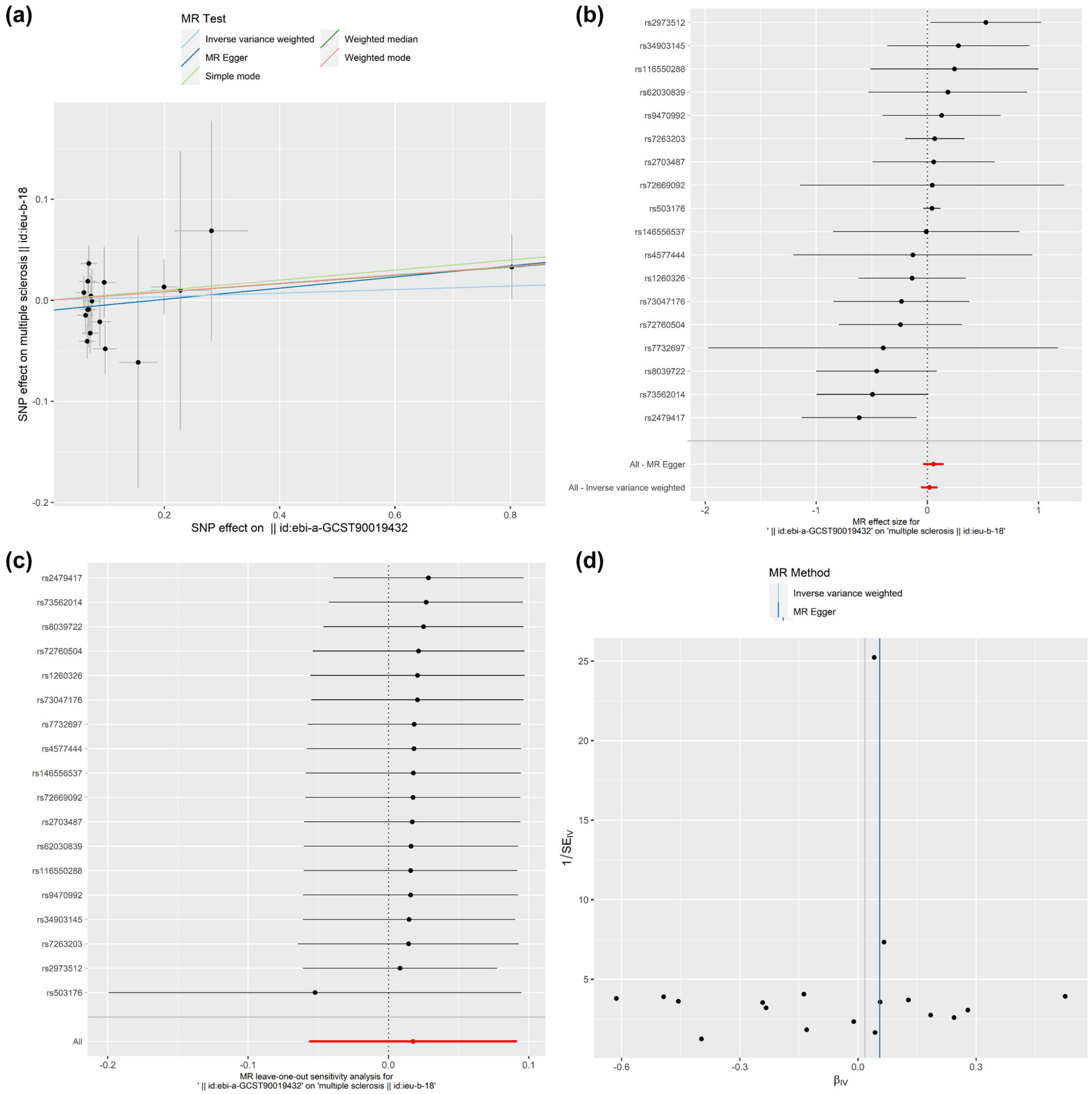

Figure S2: The causal effect of plasma FVII on MS risk. (a) Scatter plot, (b) Forest plot, (c) Leave one out plot and (d) Funnel plot. FVII, factor VII; MS, multiple sclerosis.

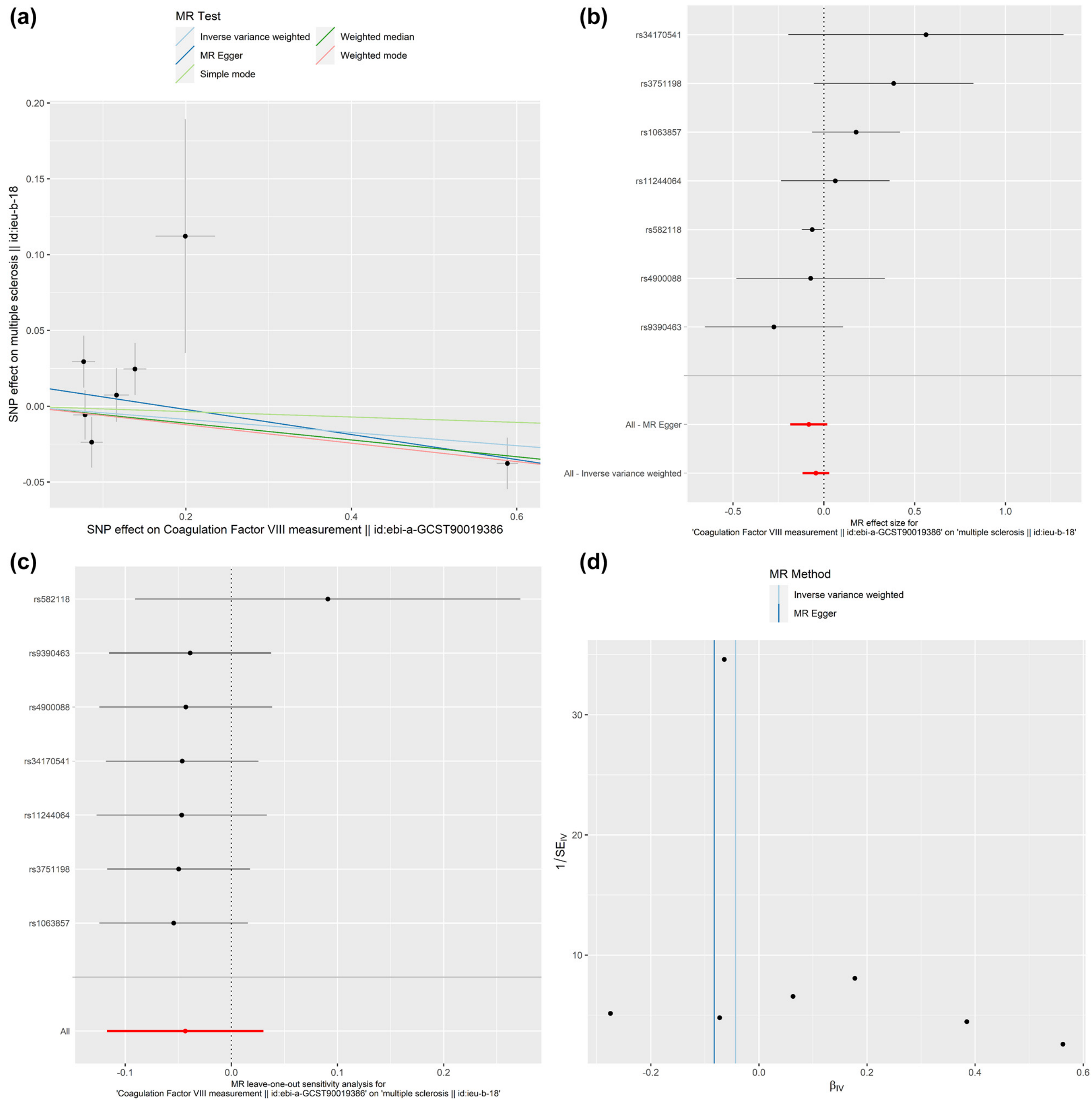

Figure S3: The causal effect of plasma FVIII on MS risk. (a) Scatter plot, (b) Forest plot, (c) Leave one out plot and (d) Funnel plot. FVIII, factor VIII; MS, multiple sclerosis.

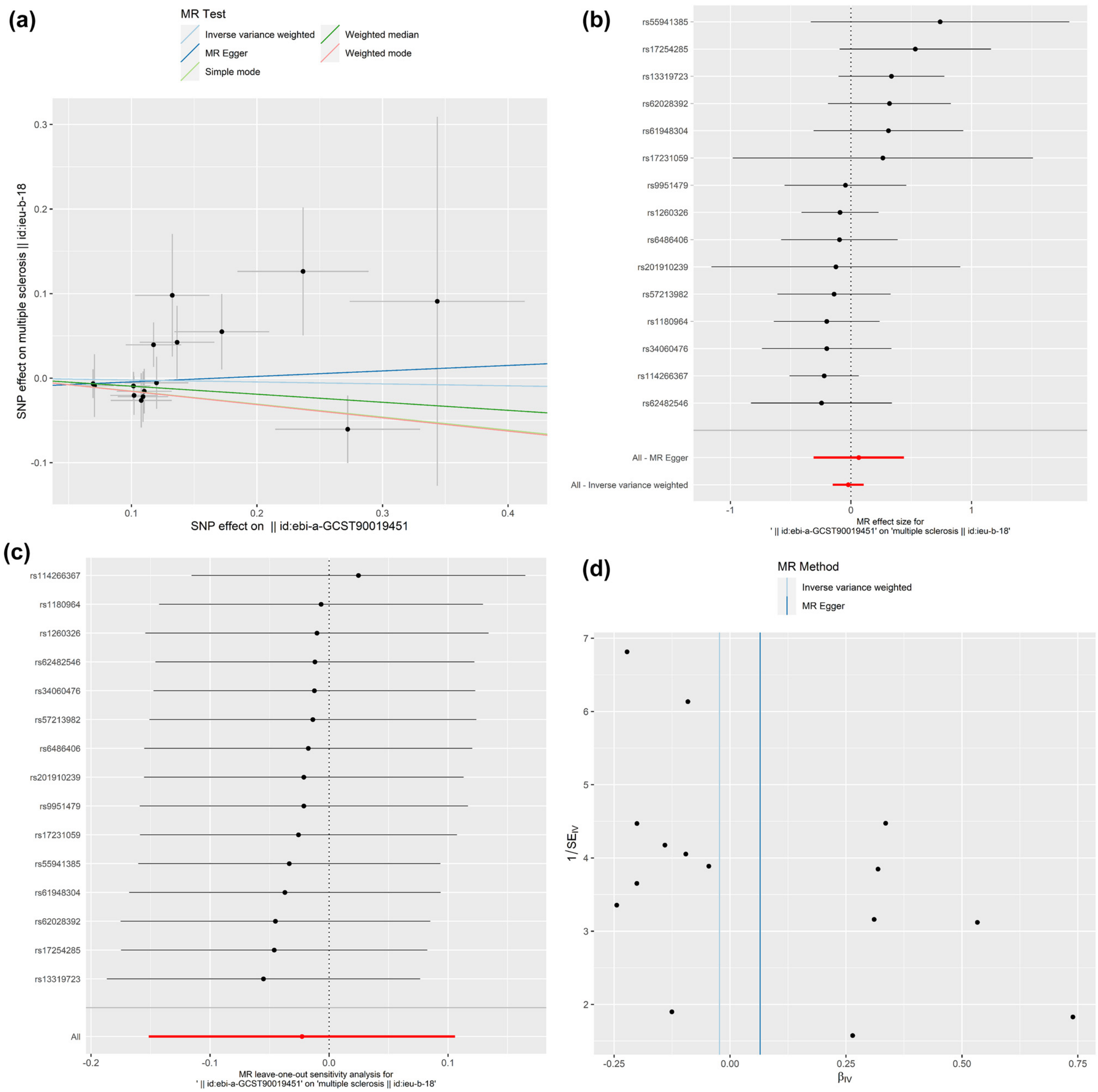

Figure S4: The causal effect of plasma FIX on MS risk. (a) Scatter plot, (b) Forest plot, (c) Leave one out plot and (d) Funnel plot. FIX, factor IX; MS, multiple sclerosis.

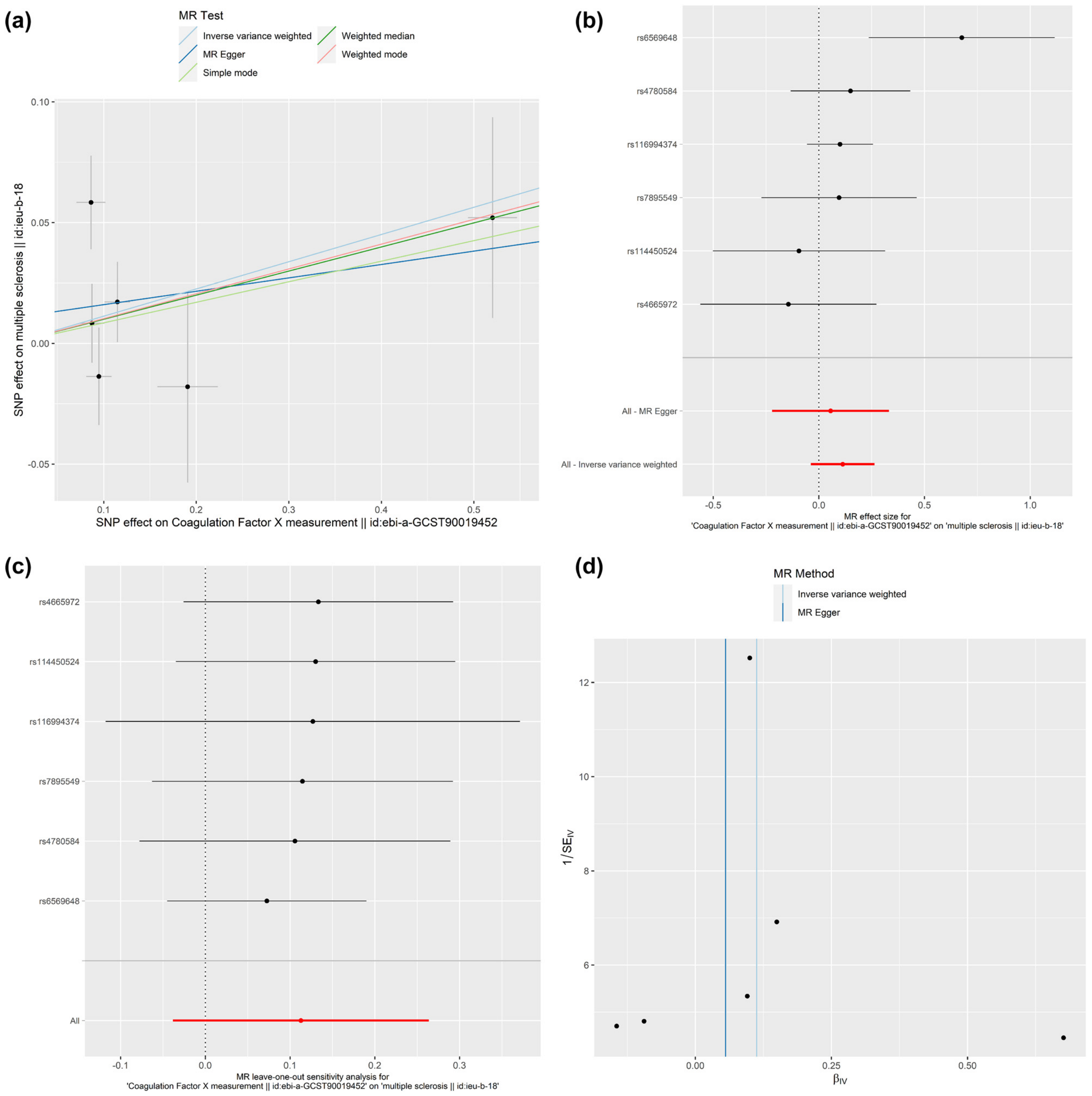

Figure S5: The causal effect of plasma FX on MS risk. (a) Scatter plot, (b) Forest plot, (c) Leave one out plot and (D) Funnel plot. FX, factor X; MS, multiple sclerosis.

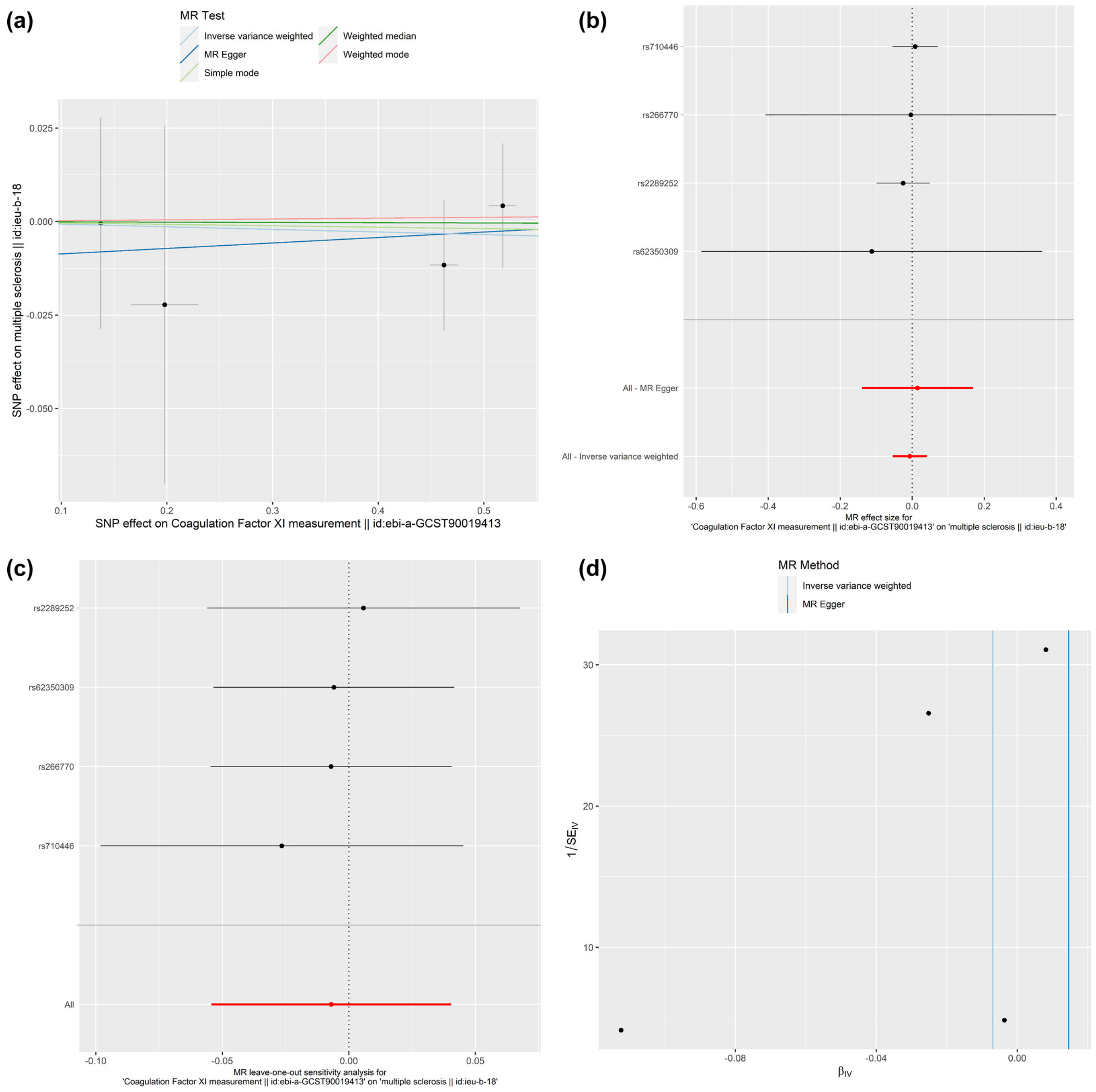

Figure S6: The causal effect of plasma FXI on MS risk. (a) Scatter plot, (b) Forest plot, (c) Leave one out plot and (d) Funnel plot. FXI, factor XI; MS, multiple sclerosis.

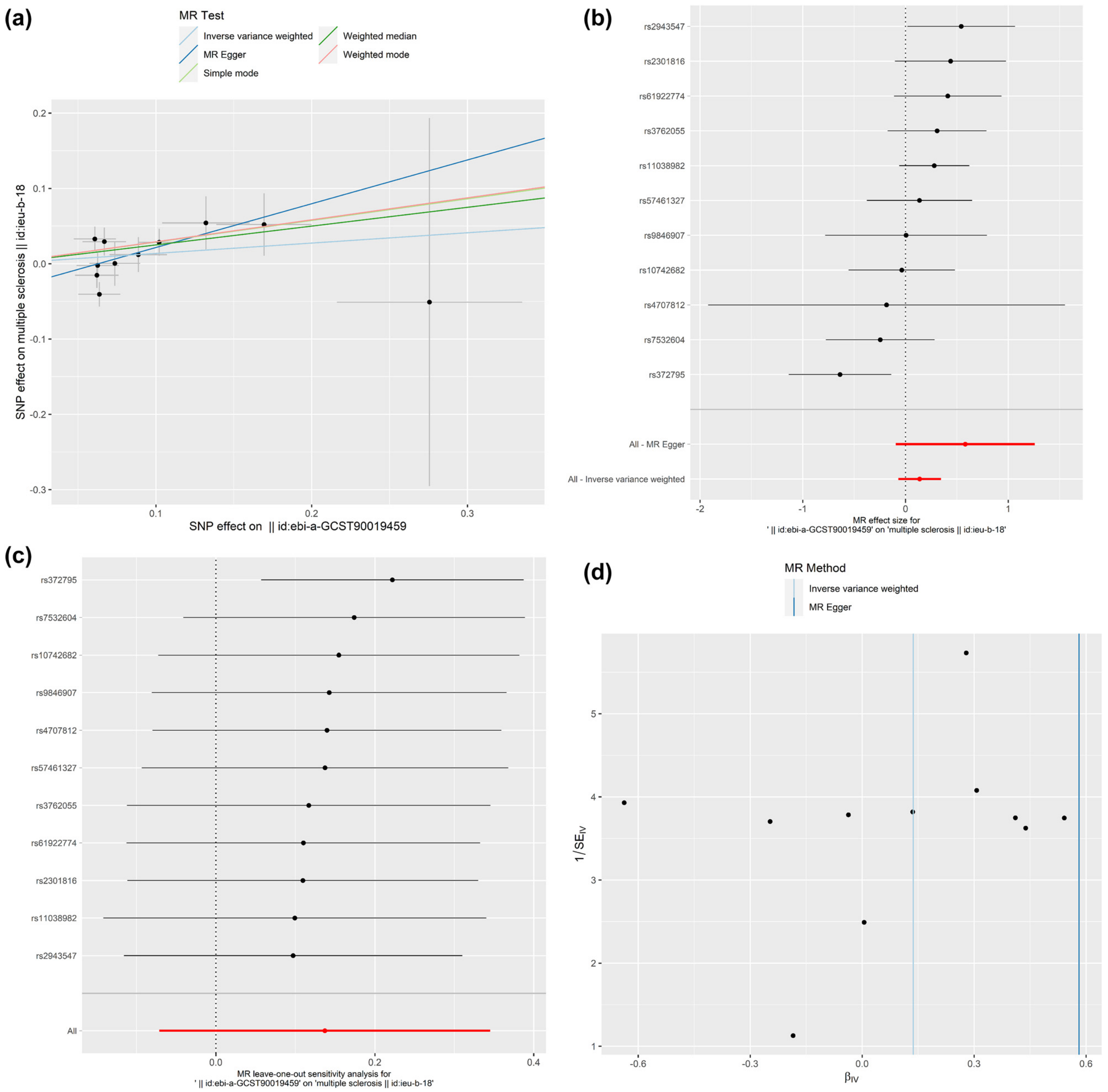

Figure S7: The causal effect of plasma prothrombin on MS risk. (a) Scatter plot, (b) Forest plot, (c) Leave one out plot and (d) Funnel plot. MS, multiple sclerosis.

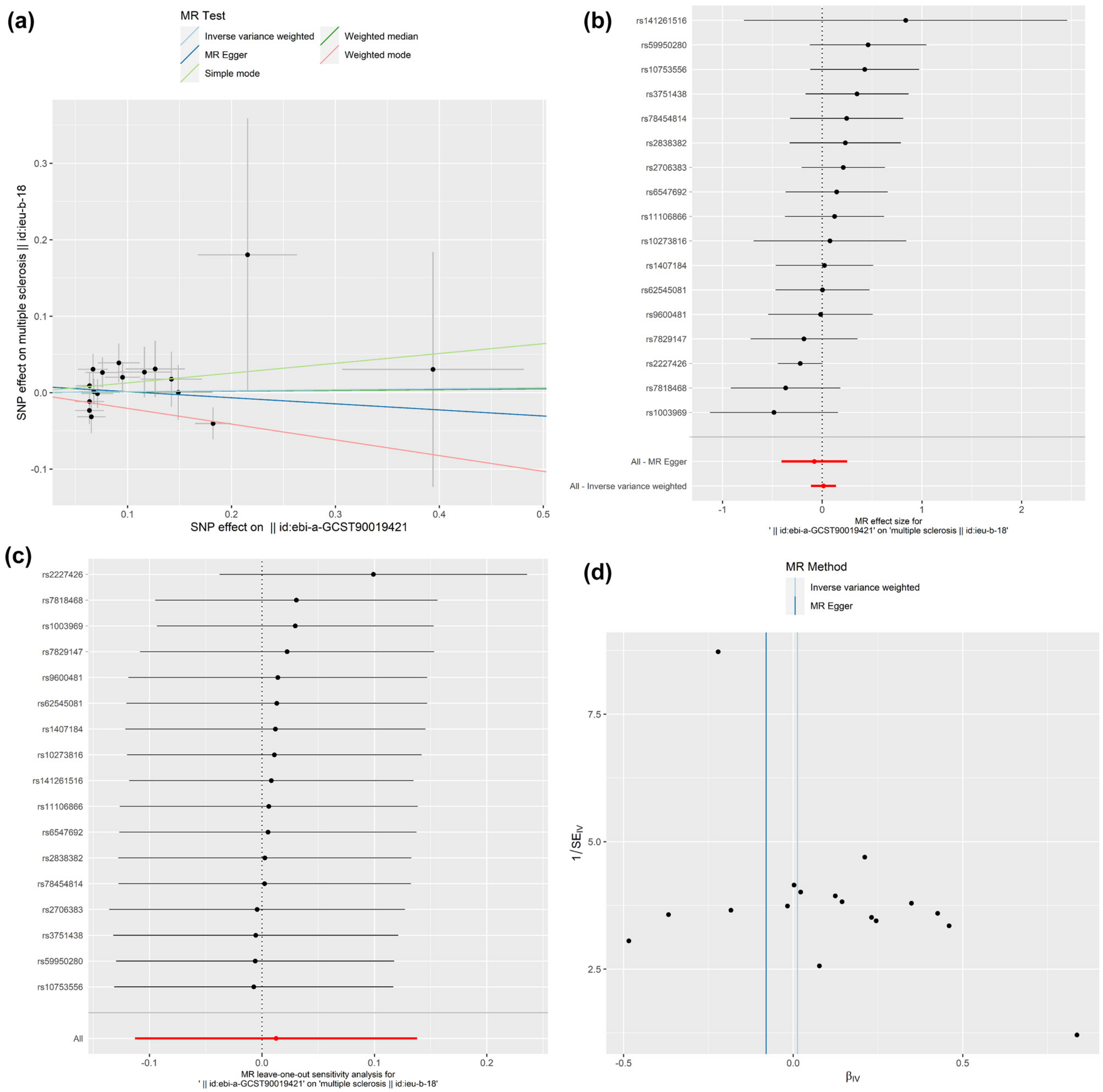

Figure S8: The causal effect of plasma fibrinogen on MS risk. (a) Scatter plot, (b) Forest plot, (c) Leave one out plot and (d) Funnel plot. MS, multiple sclerosis.

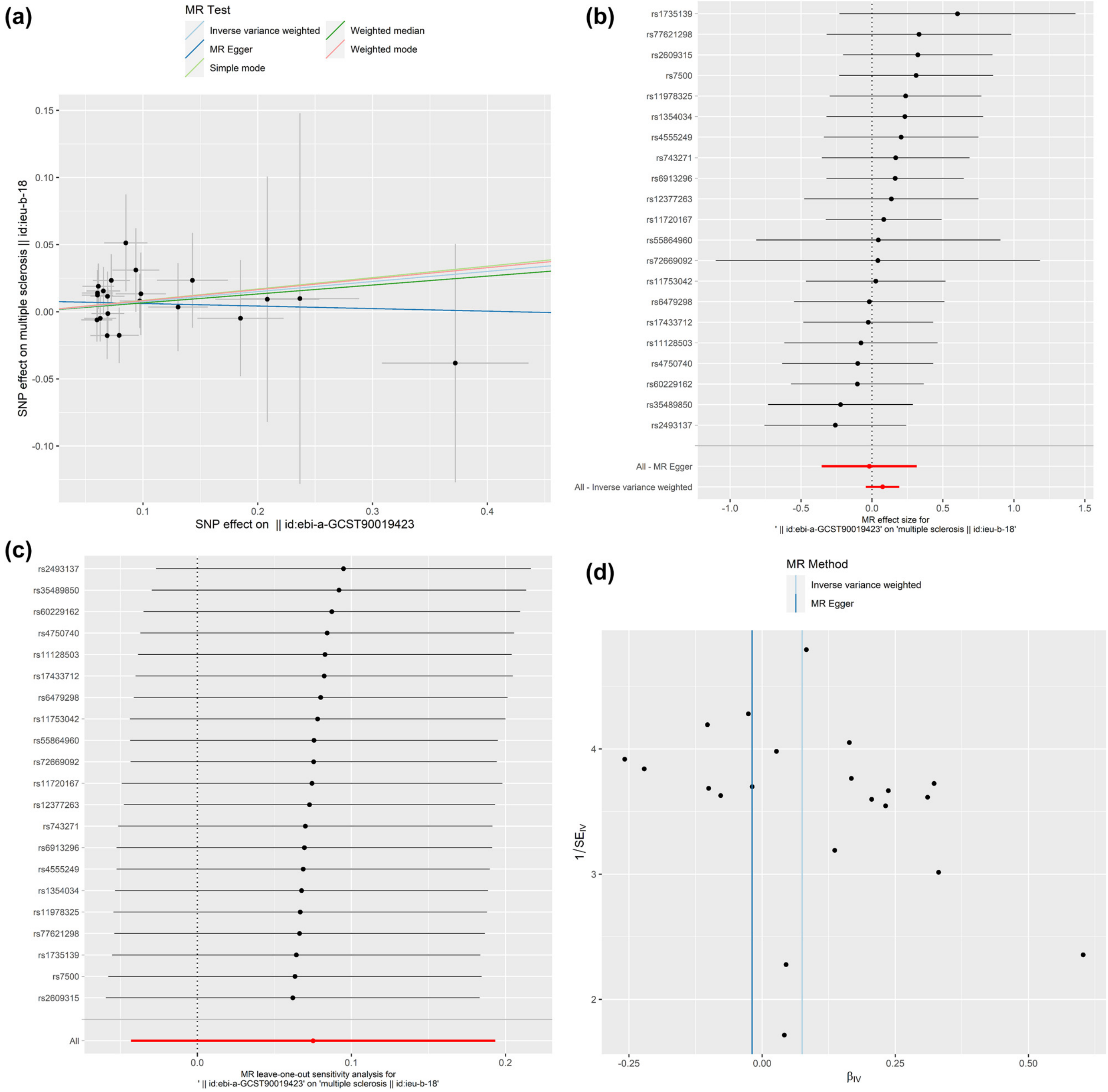

Figure S9: The causal effect of plasma protein C on MS risk. (a) Scatter plot, (b) Forest plot, (c) Leave one out plot and (d) Funnel plot. PAI-1, plasminogen activator inhibitor 1; TFPI, tissue factor pathway inhibitor; MS, multiple sclerosis.

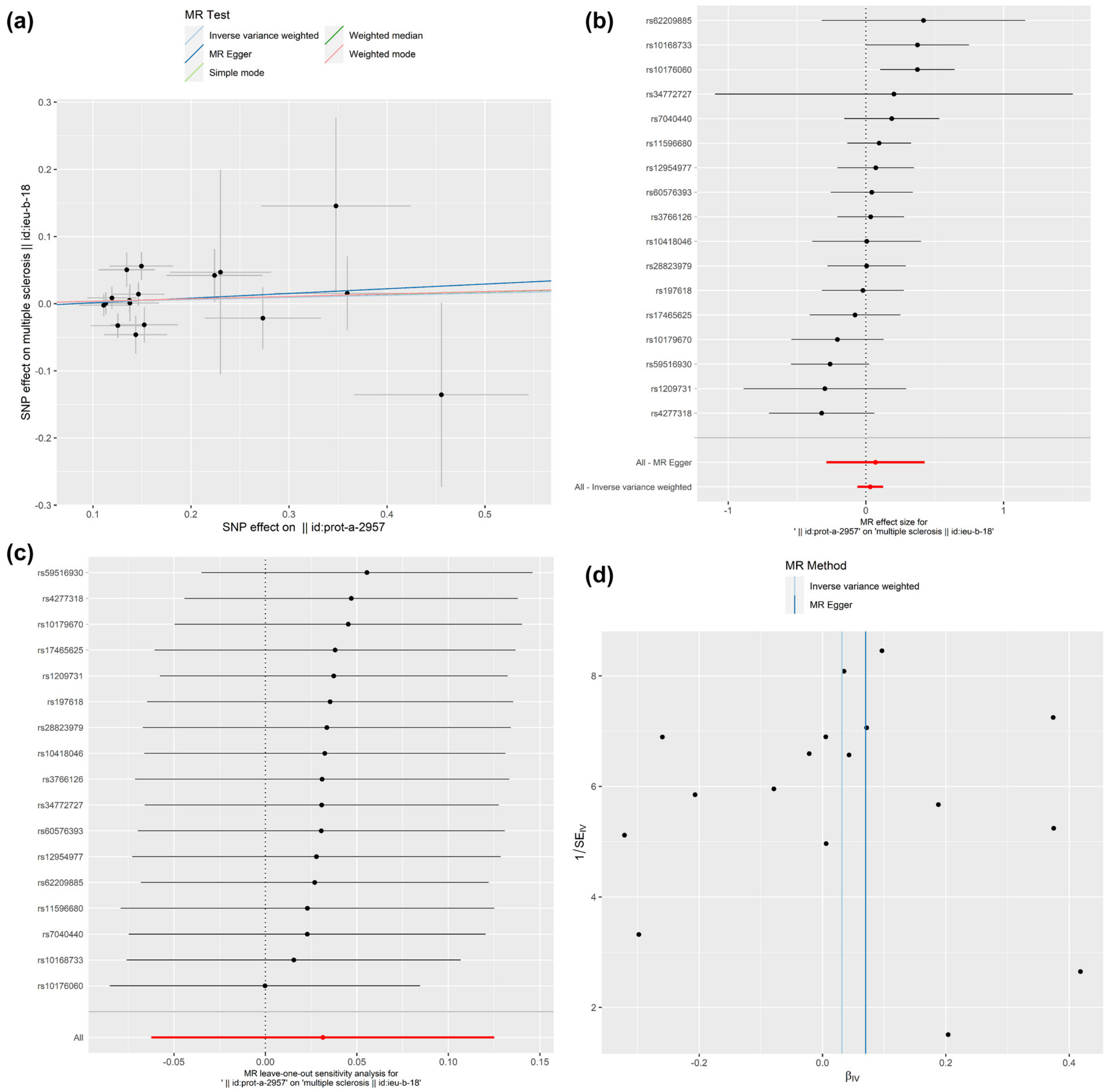

Figure S10: The causal effect of plasma TFPI on MS risk. (a) Scatter plot, (b) Forest plot, (c) Leave one out plot and (d) Funnel plot. TFPI, tissue factor pathway inhibitor; MS, multiple sclerosis.

Table S1: Details of the removed SNPs for potential horizontal pleiotropy

Exposure	SNP	Chr	Pos.	Effect allele	Other allele	Trait	Type	Related disease and traits	*P* value
FV	rs205002	6	32113312	A	G	Gene expression of PRRT1	Gene expression	Multiple sclerosis (self-reported)	1.58×10^{-15}
MS	rs2857700	6	31572481	C	T	Venous thromboembolism or von Willebrand factor levels (pleiotropy)	Diseases and traits	Venous thromboembolism	2×10^{-9}
MS	rs7975763	12	123604053	T	C	Venous thromboembolism or fibrinogen levels (pleiotropy)	Diseases and traits	Venous thromboembolism or fibrinogen levels	8×10^{-11}
MS	rs7592560	2	68647001	A	G	Venous thromboembolism or von Willebrand factor levels (pleiotropy)	Diseases and traits	Venous thromboembolism	6×10^{-10}
MS	rs7592560	2	68647001	A	G	Venous thromboembolism or factor VIII levels (pleiotropy)	Diseases and traits	Venous thromboembolism or factor VIII levels	6×10^{-10}
MS	rs7592560	2	68647001	A	G	Venous thromboembolism or factor XI levels (pleiotropy)	Diseases and traits	Venous thromboembolism or factor XI levels	6×10^{-10}
MS	rs7592560	2	68647001	A	G	Venous thromboembolism or factor VII levels (pleiotropy)	Diseases and traits	Venous thromboembolism or factor VII levels	3×10^{-10}
MS	rs7592560	2	68647001	A	G	Venous thromboembolism or fibrinogen levels (pleiotropy)	Diseases and traits	Venous thromboembolism or fibrinogen levels	4×10^{-10}

FV, factor V; MS, multiple sclerosis. SNP, single-nucleotide polymorphism; Chr, chromosome; Pos, position; The threshold was set at $P < 5\times10^{-8}$.

Table S2: Characteristics of selected SNPs for circulating hemostasis components

SNP	Trait	Chr	Pos.	Effect allele	Other allele	EAF	Beta	SE	*P value*	R^2	*F* statistic
rs12127355	TF	1	94965961	T	C	0.4584	0.0915	0.0107	1.32587×10^{-17}	0.004157	90.820458
rs1361868	TF	1	95100877	A	G	0.3045	−0.0816	0.0117	2.87×10^{-12}	0.002820	61.531905
rs11589759	TF	1	95268009	T	C	0.6163	−0.184	0.0111	6.92×10^{-62}	0.016012	354.029068
rs635634	TF	9	136155000	T	C	0.2012	−0.2212	0.0139	2.14×10^{-57}	0.015728	347.640629
rs41307428	TF	9	136336804	T	C	0.0696	0.2321	0.025	1.92×10^{-20}	0.006977	152.854734
rs6041	TF	13	113772707	A	G	0.1067	0.0996	0.0167	2.25×10^{-9}	0.001891	41.220337
rs492602	TF	19	49206417	G	A	0.4415	−0.2038	0.0101	3.92×10^{-90}	0.020483	454.945407
rs1591734	FV	1	169271902	T	C	0.1423	−0.2011	0.0194	3.08×10^{-25}	0.009872	106.740747
rs2227245	FV	1	169540080	T	C	0.3141	−0.1667	0.0145	1.35×10^{-30}	0.011974	129.744420
rs34535888	FV	6	32561527	C	T	0.1816	−0.1487	0.0180	1.34×10^{-16}	0.006573	70.831180
rs927826	FV	10	20220846	T	G	0.7117	0.1757	0.0149	5.04×10^{-32}	0.012668	137.365997
rs72760504	FVII	1	241371113	G	A	0.1305	−0.0881	0.0197	7.91×10^{-6}	0.001761	18.890993
rs2479417	FVII	1	55495744	C	T	0.6397	−0.0662	0.0139	1.78×10^{-6}	0.002020	21.671654
rs72669092	FVII	1	42176691	A	G	0.0195	0.2277	0.0511	8.24×10^{-6}	0.001983	21.268037
rs1260326	FVII	2	27730940	C	T	0.5970	−0.0673	0.0134	4.91×10^{-7}	0.002179	23.383757
rs4577444	FVII	3	184327832	C	A	0.3062	0.0688	0.0151	4.99×10^{-6}	0.002011	21.574856
rs2703487	FVII	4	55434828	T	C	0.2153	0.0728	0.0161	6.11×10^{-6}	0.001791	19.206406
rs2973512	FVII	5	11094740	T	C	0.3107	−0.0691	0.0142	1.23×10^{-6}	0.002045	21.940776
rs7732697	FVII	5	161797431	A	G	0.0475	−0.1546	0.0329	2.59×10^{-6}	0.002163	23.204654
rs9470992	FVII	6	39145552	G	A	0.5024	0.0604	0.0134	6.53×10^{-6}	0.001824	19.563836
rs73047176	FVII	7	5584193	T	C	0.341	0.0635	0.0143	9.24×10^{-6}	0.001812	19.437135
rs34903145	FVII	7	72016837	A	G	0.2831	0.0673	0.0149	6.50×10^{-6}	0.001838	19.719005
rs116550288	FVII	8	128191320	G	A	0.0115	0.2817	0.0637	9.65×10^{-6}	0.001804	19.350389
rs146556537	FVII	11	117794456	A	G	0.2327	0.0746	0.0166	7.41×10^{-6}	0.001987	21.318685
rs73562014	FVII	11	110272261	A	G	0.1228	0.0975	0.0202	1.36×10^{-6}	0.002048	21.971202
rs503176	FVII	13	113744874	G	A	0.885	0.8025	0.0197	1.00×10^{-200}	0.131087	1615.148168
rs8039722	FVII	15	92675217	C	A	0.3924	0.0714	0.0146	1.02×10^{-6}	0.002431	26.089001
rs62030839	FVII	16	17751710	T	G	0.1232	0.0957	0.0206	3.54×10^{-6}	0.001979	21.225236
rs7263203	FVII	20	33773375	C	A	0.0931	0.1995	0.0225	8.84×10^{-19}	0.006721	72.440394
rs9390463	FVIII	6	147702147	G	A	0.5002	0.0862	0.0134	1.29×10^{-10}	0.003715	39.923463
rs582118	FVIII	9	136145471	G	A	0.3362	0.5885	0.0129	1.00×10^{-200}	0.154582	1957.552628
rs11244064	FVIII	9	136161837	A	G	0.3259	0.1162	0.0152	1.75×10^{-14}	0.005933	63.894339
rs34170541	FVIII	9	135977366	G	T	0.037	−0.1994	0.036	3.16×10^{-8}	0.002833	30.420604
rs1063857	FVIII	12	6153514	G	A	0.3701	0.1385	0.0138	1.17×10^{-23}	0.008944	96.616027
rs3751198	FVIII	12	104147207	G	A	0.6072	0.0765	0.0139	3.37×10^{-8}	0.002792	29.970737
rs4900088	FVIII	14	92290681	G	A	0.5804	−0.0782	0.0142	3.87×10^{-8}	0.002979	31.983731
rs1180964	FIX	1	55218254	A	G	0.1485	−0.1021	0.0189	6.23×10^{-8}	0.002636	28.298685
rs1260326	FIX	2	27730940	C	T	0.5971	−0.1016	0.0135	4.38×10^{-14}	0.004967	53.438141

(*Continued*)

Table S2: *Continued*

SNP	Trait	Chr	Pos.	Effect allele	Other allele	EAF	Beta	SE	*P value*	R^2	*F* statistic
rs114266367	FIX	2	49817979	T	C	0.0208	−0.2723	0.0578	2.47×10^{-6}	0.003020	32.434034
rs55941385	FIX	3	141392785	T	C	0.0585	0.1325	0.0296	7.44×10^{-6}	0.001934	20.744639
rs13319723	FIX	3	177713782	C	A	0.1001	0.1176	0.0221	1.05×10^{-7}	0.002492	26.741369
rs201910239	FIX	4	79900959	A	C	0.2601	0.0706	0.0155	5.27×10^{-6}	0.001918	20.578513
rs57213982	FIX	5	6354590	T	G	0.1021	0.1102	0.0219	5.14×10^{-7}	0.002227	23.891434
rs17254285	FIX	7	39017924	G	A	0.0172	0.2367	0.0522	5.77×10^{-6}	0.001894	20.317523
rs34060476	FIX	7	73037956	G	A	0.1285	−0.1092	0.02	5.08×10^{-8}	0.002671	28.670450
rs62482546	FIX	7	107112439	G	A	0.0833	−0.1078	0.0243	9.56×10^{-6}	0.001775	19.034367
rs6486406	FIX	11	18105871	T	C	0.3518	0.0694	0.014	6.96×10^{-7}	0.002197	23.568724
rs61948304	FIX	13	47390877	T	C	0.054	0.1364	0.0297	4.45×10^{-6}	0.001901	20.389059
rs62028392	FIX	16	12513670	G	A	0.0331	0.172	0.0378	5.22×10^{-6}	0.001894	20.311727
rs9951479	FIX	18	62988255	C	A	0.0747	−0.12	0.0253	2.18×10^{-6}	0.001991	21.354445
rs17231059	FIX	21	40298249	A	G	0.0097	0.3436	0.0697	8.25×10^{-7}	0.002268	24.338187
rs4665972	FX	2	27598097	C	T	0.5968	−0.0948	0.0137	5.05×10^{-12}	0.004325	46.505647
rs114450524	FX	3	186379589	A	G	0.0461	−0.1907	0.0326	5.12×10^{-9}	0.003198	34.352130
rs6569648	FX	6	130349119	T	C	0.7605	0.0863	0.0156	3.29×10^{-8}	0.002713	29.124832
rs116994374	FX	9	117084672	A	G	0.0744	−0.5201	0.0265	1.62×10^{-85}	0.037256	414.301541
rs7895549	FX	10	65357438	A	G	0.4994	−0.0873	0.0134	8.48×10^{-11}	0.003811	40.952763
rs4780584	FX	16	16095159	T	C	0.5921	0.1147	0.0138	1.10×10^{-16}	0.006355	68.470188
rs710446	FXI	3	186459927	C	T	0.4018	0.5179	0.0129	1.00×10^{-200}	0.128937	1584.732336
rs266770	FXI	3	186545178	G	A	0.8592	−0.1373	0.0196	2.54×10^{-12}	0.004561	49.054699
rs2289252	FXI	4	187207381	T	C	0.4052	0.4622	0.0131	1.00×10^{-200}	0.102975	1229.002571
rs62350309	FXI	4	187277666	G	A	0.0502	−0.1979	0.0318	5.09×10^{-10}	0.003735	40.133747
rs7532604	prothrombin	1	171423634	T	C	0.3838	0.0621	0.014	8.78×10^{-6}	0.001824	19.564106
rs372795	prothrombin	3	158627589	G	A	0.5117	0.0637	0.0135	2.52×10^{-6}	0.002028	21.753030
rs9846907	prothrombin	3	129757554	G	T	0.7153	−0.0736	0.0163	6.69×10^{-6}	0.002206	23.672700
rs4707812	prothrombin	6	94544549	T	C	0.0146	0.2756	0.0595	3.58×10^{-6}	0.002186	23.449375
rs2943547	prothrombin	8	76451098	G	A	0.5207	−0.0608	0.0135	6.76×10^{-6}	0.001845	19.790715
rs3762055	prothrombin	9	117090575	C	T	0.0684	−0.1694	0.0306	2.98×10^{-8}	0.003657	39.297129
rs11038982	prothrombin	11	46762419	A	C	0.519	−0.1021	0.0136	5.93×10^{-14}	0.005205	56.012818
rs10742682	prothrombin	11	43665857	T	C	0.4481	0.0626	0.0136	4.41×10^{-6}	0.001938	20.791405
rs61922774	prothrombin	12	54111758	A	G	0.0689	0.1321	0.0281	2.55×10^{-6}	0.002239	24.024363
rs57461327	prothrombin	17	5014416	A	G	0.1605	−0.0887	0.0184	1.44×10^{-6}	0.002120	22.746887
rs2301816	prothrombin	22	31266546	C	T	0.6134	0.0669	0.014	1.84×10^{-6}	0.002123	22.773928
rs10753556	fibrinogen	1	23732762	G	A	0.8738	0.0916	0.0201	5.28×10^{-6}	0.001851	19.848331
rs6547692	fibrinogen	2	27734972	A	G	0.5469	0.0634	0.0133	2.03×10^{-6}	0.001992	21.369962
rs59950280	fibrinogen	4	3452345	A	G	0.3419	0.0667	0.0144	3.34×10^{-6}	0.002002	21.476833
rs2227426	fibrinogen	4	155493171	A	G	0.1787	0.1823	0.0172	3.72×10^{-26}	0.009755	105.466446
rs2706383	fibrinogen	5	131792402	A	G	0.1959	−0.0952	0.0169	1.62×10^{-8}	0.002855	30.656137

(*Continued*)

Table S2: *Continued*

SNP	Trait	Chr	Pos.	Effect allele	Other allele	EAF	Beta	SE	*P value*	R^2	*F* statistic
rs1407184	fibrinogen	6	117733717	A	G	0.3773	−0.0675	0.0143	2.33×10^{-6}	0.002141	22.970013
rs78454814	fibrinogen	6	52229848	T	C	0.0601	0.1266	0.0285	8.59×10^{-6}	0.001811	19.420834
rs10273816	fibrinogen	7	132785591	C	A	0.0061	0.3939	0.0874	6.51×10^{-6}	0.001881	20.179926
rs7818468	fibrinogen	8	29731466	G	A	0.3713	−0.0633	0.0138	4.54×10^{-6}	0.001871	20.065328
rs7829147	fibrinogen	8	14905962	A	G	0.3488	−0.0634	0.014	6.27×10^{-6}	0.001826	19.584855
rs62545081	fibrinogen	9	28707073	A	C	0.0437	0.1488	0.0327	5.50×10^{-6}	0.001851	19.849206
rs11106866	fibrinogen	12	93516373	T	C	0.0559	−0.1422	0.0295	1.45×10^{-6}	0.002134	22.898875
rs9600481	fibrinogen	13	76068181	G	A	0.2481	−0.071	0.0154	3.69×10^{-6}	0.001881	20.173365
rs3751438	fibrinogen	13	44600891	C	T	0.7918	0.0758	0.0163	3.33×10^{-6}	0.001894	20.319610
rs141261516	fibrinogen	15	66688150	G	A	0.0212	0.2155	0.0477	6.20×10^{-6}	0.001927	20.673758
rs2838382	fibrinogen	21	45218645	T	C	0.0694	0.1162	0.026	7.78×10^{-6}	0.001744	18.704673
rs1003969	fibrinogen	22	50709987	C	T	0.4361	0.0652	0.0138	2.24×10^{-6}	0.002091	22.431049
rs72669092	protein C	1	42176691	A	G	0.0195	0.2367	0.0515	4.35×10^{-6}	0.002142	22.986212
rs60229162	protein C	1	52026593	A	G	0.0116	−0.3721	0.0638	5.56×10^{-9}	0.003175	34.099528
rs2493137	protein C	1	230852116	C	T	0.2975	−0.0688	0.0146	2.40×10^{-6}	0.001979	21.224014
rs1354034	protein C	3	56849749	C	T	0.6001	−0.0604	0.0136	9.35×10^{-6}	0.001751	18.778774
rs11128503	protein C	3	76163422	T	C	0.3828	−0.0628	0.014	6.84×10^{-6}	0.001864	19.988696
rs11720167	protein C	3	165486145	G	T	0.206	−0.0971	0.0173	2.00×10^{-8}	0.003084	33.122643
rs11753042	protein C	6	135142809	A	G	0.0757	0.1306	0.0259	4.66×10^{-7}	0.002387	25.614758
rs6913296	protein C	6	91124239	G	A	0.0511	−0.1431	0.0309	3.53×10^{-6}	0.001986	21.303019
rs2609315	protein C	6	168819212	A	G	0.2235	0.0724	0.0162	7.35×10^{-6}	0.001819	19.513907
rs11978325	protein C	7	24380815	A	G	0.6983	0.0655	0.0147	8.31×10^{-6}	0.001808	19.388445
rs6479298	protein C	9	107952654	G	A	0.6711	−0.0694	0.0143	1.22×10^{-6}	0.002126	22.811385
rs12377263	protein C	9	132937021	T	C	0.1148	−0.0982	0.0219	7.34×10^{-6}	0.001960	21.024008
rs7500	protein C	10	12877510	T	C	0.3656	0.0611	0.0138	9.67×10^{-6}	0.001732	18.572132
rs17433712	protein C	10	90500634	T	C	0.0337	0.185	0.0376	8.61×10^{-7}	0.002229	23.917276
rs4750740	protein C	10	130928107	C	A	0.5026	0.0599	0.0135	9.18×10^{-6}	0.001794	19.240615
rs743271	protein C	14	24548567	A	G	0.6738	−0.0691	0.0148	3.12×10^{-6}	0.002099	22.518570
rs55864960	protein C	17	52914119	A	G	0.0263	−0.2083	0.0453	4.19×10^{-6}	0.002222	23.844206
rs35489850	protein C	17	6953103	C	T	0.2088	−0.0793	0.017	2.93×10^{-6}	0.002078	22.290698
rs4555249	protein C	18	61136536	A	C	0.4575	−0.0604	0.0135	8.19×10^{-6}	0.001811	19.422679
rs77621298	protein C	20	5023173	G	A	0.1354	0.0938	0.0205	4.59×10^{-6}	0.002060	22.100005
rs1735139	protein C	21	41105579	C	A	0.1777	0.0851	0.0188	6.11×10^{-6}	0.002116	22.706710
rs3766126	TFPI	1	169575057	T	G	0.3281	−0.1373	0.0265	2.24×10^{-7}	0.008312	27.649609
rs1209731	TFPI	1	169324793	C	T	0.97726	0.4555	0.089	3.09×10^{-7}	0.009222	30.705285
rs197618	TFPI	1	57686541	G	A	0.58479	−0.111	0.025	9.12×10^{-6}	0.005983	19.857856
rs10168733	TFPI	2	132581937	T	C	0.27779	−0.1345	0.0289	3.24×10^{-6}	0.007259	24.121309
rs10176060	TFPI	2	154195035	C	A	0.18343	0.1495	0.0324	3.80×10^{-6}	0.006695	22.236992
rs10179670	TFPI	2	36247273	C	T	0.15164	0.1524	0.0344	9.77×10^{-6}	0.005976	19.832581

(*Continued*)

Table S2: *Continued*

SNP	Trait	Chr	Pos.	Effect allele	Other allele	EAF	Beta	SE	*P value*	R^2	*F* statistic
rs60576393	TFPI	4	180569539	T	C	0.03082	0.3594	0.0749	1.58×10^{-6}	0.007717	25.654868
rs28823979	TFPI	7	121288416	G	A	0.38693	0.1131	0.0254	8.91×10^{-6}	0.006069	20.142976
rs7040440	TFPI	9	117091074	T	C	0.08104	−0.2241	0.0488	4.47×10^{-6}	0.007480	24.862962
rs59516930	TFPI	9	132305715	T	G	0.32384	0.1254	0.0275	5.25×10^{-6}	0.006887	22.876444
rs11596680	TFPI	10	20208483	G	A	0.3283	0.1464	0.0266	3.80×10^{-8}	0.009453	31.482231
rs17465625	TFPI	10	8293735	C	T	0.05038	0.2734	0.0594	4.17×10^{-6}	0.007152	23.764826
rs34772727	TFPI	14	60053085	A	G	0.06029	0.2301	0.0516	8.32×10^{-6}	0.005999	19.911226
rs4277318	TFPI	15	40605497	G	A	0.79715	0.1437	0.032	7.24×10^{-6}	0.006678	22.179458
rs12954977	TFPI	18	4205510	C	T	0.49093	0.1194	0.0252	2.19×10^{-6}	0.007126	23.676845
rs10418046	TFPI	19	54327869	G	T	0.21727	−0.1377	0.0299	4.07×10^{-6}	0.006449	21.414201
rs62209885	TFPI	20	39327519	C	A	0.03498	−0.3479	0.0763	5.13×10^{-6}	0.008171	27.179449

TF, tissue factor; F(V, VII, VIII, IX, X, XI), factor (V, VII, VIII, IX, X, XI); TFPI, tissue factor pathway inhibitor. Chr, chromosome; EAF, Effect allele frequency; Pos, position; SE, standard error; SNP, single-nucleotide polymorphism. The threshold was set at $P < 5\times10^{-8}$.

Table S3: Characteristics of selected SNPs for multiple sclerosis

SNP	Trait	Chr	Pos.	Effect allele	Other allele	EAF	Beta	SE	*P* value	R^2	*F* statistic
rs10063294	MS	5	35877505	A	G	0.5577	−0.09905	0.016264	1.13×10^{-9}	0.00484	563.1853
rs1014486	MS	3	159691112	C	T	0.4592	0.10508	0.016368	1.36×10^{-10}	0.005484	638.5711
rs1026916	MS	17	40529835	G	A	0.6342	−0.12965	0.017431	1.02×10^{-13}	0.007799	910.2861
rs1077667	MS	19	6668972	T	C	0.2286	−0.15186	0.021225	8.37×10^{-13}	0.008134	949.6055
rs10801908	MS	1	117090493	T	C	0.1382	−0.21495	0.026363	3.54×10^{-16}	0.011006	1288.659
rs1112718	MS	10	94479107	G	A	0.3926	−0.10562	0.016688	2.46×10^{-10}	0.00532	619.4076
rs11256593	MS	10	6117322	T	C	0.5547	0.186314	0.017352	6.78×10^{-27}	0.017149	2020.488
rs114872782	MS	6	32241452	T	C	0.01889	−2.45044	0.255159	7.72×10^{-22}	0.222571	33152.72
rs11666263	MS	19	10590684	G	A	0.3091	−0.10283	0.018039	1.2×10^{-8}	0.004516	525.3353
rs11711621	MS	3	169524016	T	C	0.2425	−0.10427	0.019202	5.63×10^{-8}	0.003994	464.4015
rs11749040	MS	5	40396425	A	G	0.1352	0.196745	0.023346	3.54×10^{-17}	0.009052	1057.77
rs1177228	MS	2	61242410	G	A	0.7555	0.107418	0.018659	8.57×10^{-9}	0.004263	495.7526
rs11809700	MS	1	93152635	T	C	0.2853	0.144448	0.018351	3.51×10^{-15}	0.008509	993.8072
rs12147246	MS	14	103265844	G	A	0.6551	−0.09938	0.016923	4.29×10^{-9}	0.004463	519.1222
rs12365699	MS	11	118743286	A	G	0.165	−0.14375	0.022849	3.15×10^{-10}	0.005694	663.1819
rs1250551	MS	10	81059335	T	G	0.3111	0.115748	0.017369	2.66×10^{-11}	0.005743	668.8469
rs12612620	MS	2	112488876	A	G	0.2247	0.211833	0.036063	4.26×10^{-9}	0.015635	1839.273
rs12925972	MS	16	79111297	C	T	0.5328	0.094583	0.017081	3.07×10^{-8}	0.004454	518.0485
rs13327021	MS	3	27783015	T	C	0.3797	0.115186	0.017122	1.73×10^{-11}	0.00625	728.2943
rs140522	MS	22	50971266	C	T	0.6799	−0.1106	0.017536	2.85×10^{-10}	0.005324	619.8266
rs1465697	MS	19	49837246	T	C	0.2376	0.124317	0.018766	3.48×10^{-11}	0.005599	652.0354
rs17124032	MS	14	88546009	A	G	0.08151	−0.2168	0.031622	7.08×10^{-12}	0.007038	820.7777
rs1738074	MS	6	159465977	C	T	0.5666	0.113729	0.016706	9.91×10^{-12}	0.006352	740.3172
rs17724508	MS	16	79350204	C	T	0.04076	−0.20807	0.038241	5.3×10^{-8}	0.003385	393.3686
rs1860545	MS	12	6446777	A	G	0.4225	0.116534	0.017031	7.79×10^{-12}	0.006627	772.5275
rs2150879	MS	17	57859210	A	G	0.5567	−0.10355	0.016477	3.29×10^{-10}	0.005292	616.109
rs2248461	MS	20	52792202	A	G	0.3817	−0.10814	0.017417	5.33×10^{-10}	0.00552	642.7713
rs2317231	MS	1	157686337	T	G	0.4672	−0.10057	0.016745	1.9×10^{-9}	0.005035	586.0436
rs2546890	MS	5	158759900	G	A	0.5089	−0.11698	0.016418	1.04×10^{-12}	0.00684	797.5743
rs2681424	MS	3	121769522	C	T	0.506	−0.12116	0.016578	2.71×10^{-13}	0.007338	856.054
rs28703878	MS	8	79417222	G	A	0.3201	0.133646	0.021434	4.51×10^{-10}	0.007775	907.3494
rs34695601	MS	14	76014298	C	T	0.2495	−0.10948	0.019791	3.16×10^{-8}	0.004489	522.1591
rs354033	MS	7	149289464	A	G	0.2604	−0.10796	0.018943	1.21×10^{-8}	0.004489	522.1988
rs35486093	MS	1	85729820	G	A	0.08648	0.179486	0.028064	1.6×10^{-10}	0.00509	592.4524
rs35540610	MS	2	231121829	C	T	0.2117	0.135143	0.019352	2.88×10^{-12}	0.006096	710.2271
rs35703946	MS	16	86021505	A	G	0.1372	−0.17252	0.02874	1.94×10^{-9}	0.007047	821.8202
rs3809627	MS	16	30103160	A	C	0.4165	−0.09695	0.01754	3.25×10^{-8}	0.004569	531.491
rs415759	MS	16	1066917	C	T	0.1889	0.118784	0.021756	4.76×10^{-8}	0.004324	502.8592
rs4325907	MS	3	101749022	T	C	0.6531	−0.09927	0.016831	3.68×10^{-9}	0.004465	519.3837
rs438613	MS	3	28072086	C	T	0.4871	0.138021	0.016606	9.43×10^{-17}	0.009519	1112.851

(*Continued*)

Table S3: *Continued*

SNP	Trait	Chr	Pos.	Effect allele	Other allele	EAF	Beta	SE	*P* value	R^2	*F* statistic
rs478093	MS	1	120255126	G	A	0.6859	0.105138	0.017904	4.3×10^{-9}	0.004763	554.1967
rs4947255	MS	6	32207483	T	C	0.03777	−0.55222	0.055504	2.54×10^{-23}	0.022165	2624.958
rs55970742	MS	7	2441337	T	C	0.6789	−0.10037	0.017899	2.05×10^{-8}	0.004392	510.8978
rs56232455	MS	11	321235	A	G	0.4433	0.15841	0.028125	1.78×10^{-8}	0.012386	1452.242
rs58546351	MS	6	32483611	G	A	0.0338	−1.89446	0.053176	1×10^{-200}	0.234415	35457.1
rs59655222	MS	1	200875897	C	T	0.2734	−0.12319	0.018628	3.76×10^{-11}	0.00603	702.4585
rs6032662	MS	20	44734310	T	C	0.7425	−0.13383	0.018329	2.85×10^{-13}	0.006849	798.572
rs62420820	MS	6	137438057	A	G	0.2276	0.137237	0.018751	2.5×10^{-13}	0.006622	771.9421
rs6496663	MS	15	90887584	C	A	0.3141	0.100594	0.018109	2.78×10^{-8}	0.00436	507.1226
rs6670198	MS	1	2520527	C	T	0.333	−0.14503	0.017642	2.03×10^{-16}	0.009343	1092.146
rs6763437	MS	3	119145390	A	G	0.04175	−0.77914	0.132145	3.72×10^{-9}	0.048573	5911.999
rs6990534	MS	8	128814091	G	A	0.669	0.10714	0.018154	3.6×10^{-9}	0.005084	591.7158
rs701006	MS	12	58106836	G	A	0.6074	0.113864	0.016836	1.35×10^{-11}	0.006183	720.5001
rs7190580	MS	16	11403470	G	A	0.7346	−0.09803	0.01794	4.64×10^{-8}	0.003747	435.5876
rs7200146	MS	16	11213449	T	G	0.6292	−0.17079	0.016949	7×10^{-24}	0.01361	1597.854
rs72928038	MS	6	90976768	A	G	0.1799	0.160521	0.024761	9.01×10^{-11}	0.007603	887.194
rs743771	MS	6	32976909	A	C	0.4851	−0.09939	0.016203	8.55×10^{-10}	0.004935	574.3201
rs74449127	MS	1	101290432	G	A	0.2654	−0.19696	0.02558	1.36×10^{-14}	0.015127	1778.638
rs7855251	MS	9	100868189	C	T	0.2843	−0.11011	0.02009	4.23×10^{-8}	0.004934	574.1744
rs9277647	MS	6	33083750	T	C	0.1829	−0.21381	0.021168	5.47×10^{-24}	0.013664	1604.245
rs9591325	MS	13	50811220	C	T	0.05467	−0.21237	0.033989	4.16×10^{-10}	0.004662	542.343
rs9610458	MS	22	22205353	T	C	0.5417	0.114221	0.01651	4.57×10^{-12}	0.006478	755.032
rs9955954	MS	18	56348044	G	A	0.2207	−0.11004	0.019451	1.54×10^{-8}	0.004165	484.3367
rs9992763	MS	4	109058718	T	G	0.5577	−0.09003	0.016461	4.51×10^{-8}	0.003999	464.9585

MS, multiple sclerosis. Chr, chromosome; EAF, Effect allele frequency; Pos, position; SE, standard error; SNP, single-nucleotide polymorphism. The threshold was set at $P < 1\times10^{-7}$.

Table S4: Reverse causal relationship of circulating hemostasis components with multiple sclerosis

Outcome	nSNPs	Method	OR (95%CI)	*P* value
Plasma TF	64	IVW	0.995 (0.978–1.013)	0.592
		MR Egger	0.996 (0.970–1.022)	0.739
		Weighted Median	0.991 (0.970–1.014)	0.447
		Simple mode	1.033 (0.979–1.090)	0.238
		Weighted mode	0.994 (0.971–1.018)	0.635
		MR-PRESSO	0.995 (0.979–1.011)	0.554
Plasma FV	64	IVW	1.006 (0.985–1.028)	0.572
		MR Egger	1.003 (0.972–1.035)	0.830
		Weighted Median	1.015 (0.982–1.048)	0.375
		Simple mode	0.977 (0.910–1.049)	0.521
		Weighted mode	1.008 (0.975–1.042)	0.623
		MR-PRESSO	1.017 (1.000–1.035)	0.054
Plasma FVII	64	IVW	1.008 (0.988–1.029)	0.425
		MR Egger	0.998 (0.968–1.029)	0.903
		Weighted Median	1.004 (0.972–1.037)	0.815
		Simple mode	1.024 (0.959–1.093)	0.479
		Weighted mode	1.007 (0.978–1.037)	0.652
		MR-PRESSO	1.000 (0.985–1.016)	0.988
Plasma FVIII	64	IVW	0.985 (0.965–1.006)	0.156
		MR Egger	0.981 (0.951–1.012)	0.236
		Weighted Median	0.989 (0.959–1.020)	0.487
		Simple mode	1.005 (0.942–1.073)	0.877
		Weighted mode	0.984 (0.954–1.014)	0.298
		MR-PRESSO	0.992 (0.975–1.009)	0.348
Plasma FIX	64	IVW	1.002 (0.979–1.026)	0.849
		MR Egger	0.987 (0.954–1.021)	0.454
		Weighted Median	0.995 (0.964–1.027)	0.738
		Simple mode	0.982 (0.919–1.049)	0.588
		Weighted mode	0.990 (0.959–1.022)	0.548
		MR-PRESSO	0.998 (0.979–1.019)	0.879
Plasma FX	64	IVW	1.007 (0.986–1.028)	0.521
		MR Egger	1.008 (0.977–1.040)	0.607
		Weighted Median	1.008 (0.978–1.040)	0.589
		Simple mode	0.992 (0.933–1.055)	0.795
		Weighted mode	1.005 (0.973–1.039)	0.744
		MR-PRESSO	0.995 (0.979–1.012)	0.585
Plasma FXI	64	IVW	1.009 (0.986–1.032)	0.455
		MR Egger	1.000 (0.967–1.035)	0.993
		Weighted Median	1.020 (0.984–1.058)	0.282
		Simple mode	1.044 (0.950–1.147)	0.374

(*Continued*)

Table S4: *Continued*

Outcome	nSNPs	Method	OR (95%CI)	*P* value
		Weighted mode	1.012 (0.975–1.051)	0.529
		MR-PRESSO	1.002 (0.983–1.022)	0.821
Plasma prothrombin	64	IVW	1.001 (0.979–1.023)	0.918
		MR Egger	1.005 (0.973–1.039)	0.754
		Weighted Median	1.010 (0.978–1.042)	0.544
		Simple mode	1.008 (0.937–1.084)	0.840
		Weighted mode	1.004 (0.976–1.033)	0.771
		MR-PRESSO	1.002 (0.983–1.021)	0.855
Plasma fibrinogen	64	IVW	0.984 (0.962–1.006)	0.142
		MR Egger	0.992 (0.960–1.025)	0.643
		Weighted Median	0.989 (0.956–1.024)	0.540
		Simple mode	0.985 (0.912–1.065)	0.711
		Weighted mode	0.987 (0.956–1.019)	0.422
		MR-PRESSO	0.991 (0.972–1.011)	0.377
Plasma PC	64	IVW	1.013 (0.992–1.034)	0.231
		MR Egger	1.012 (0.981–1.043)	0.469
		Weighted Median	1.008 (0.978–1.039)	0.595
		Simple mode	0.995 (0.936–1.059)	0.886
		Weighted mode	1.010 (0.977–1.043)	0.558
		MR-PRESSO	1.012 (0.996–1.028)	0.138
Plasma TFPI	64	IVW	0.980 (0.938–1.024)	0.371
		MR Egger	1.000 (0.931–1.075)	0.993
		Weighted Median	0.999 (0.934–1.069)	0.976
		Simple mode	0.990 (0.860–1.140)	0.894
		Weighted mode	0.997 (0.933–1.065)	0.924
		MR-PRESSO	0.992 (0.955–1.031)	0.696

Abbreviations: nSNPs, number of single nucleotide polymorphisms; TF, tissue factor; F(V, VII, VIII, IX, X, XI), factor (V, VII, VIII, IX, X, XI); PC, protein C;TFPI, tissue factor pathway inhibitor; IVW, inverse-variance weighted; MR-PRESSO, Pleiotropy Residual Sum and Outlier; OR: odds ratio; CI, confdence interval.
